# In Vitro Engineered ECM‐incorporated Hydrogels for Osteochondral Tissue Repair: A Cell‐Free Approach

**DOI:** 10.1002/adhm.202402701

**Published:** 2025-01-05

**Authors:** Ali Coyle, Aishik Chakraborty, Jiaqi Huang, Yasmeen Shamiya, Wei Luo, Arghya Paul

**Affiliations:** ^1^ School of Biomedical Engineering The University of Western Ontario London ON N6A 5B9 Canada; ^2^ Department of Chemical and Biochemical Engineering The University of Western Ontario London ON N6A 5B9 Canada; ^3^ Collaborative Specialization in Musculoskeletal Health Research and Bone and Joint Institute The University of Western Ontario London ON N6A 5B9 Canada; ^4^ Department of Chemistry The University of Western Ontario London ON N6A 5B9 Canada

**Keywords:** 3D printing, bone regeneration, cartilage regeneration, cell‐derived extracellular matrix (ECM), mesenchymal stem cells

## Abstract

Prevalence of osteoarthritis has been increasing in aging populations, which has necessitated the use of advanced biomedical treatments. These involve grafts or delivering drug molecules entrapped in scaffolds. However, such treatments often show suboptimal therapeutic effects due to poor half‐life and off‐target effects of drug molecules. As a countermeasure, a 3D printable robust hydrogel‐based tissue‐repair platform is developed containing decellularized extracellular matrix (dECM) from differentiated mammalian cells as the therapeutic cargo. Here, pre‐osteoblastic and pre‐chondrogenic murine cells are differentiated in vitro, decellularized, and incorporated into methacrylated gelatin (GelMA) solutions to form osteogenic (GelO) and chondrogenic (GelC) hydrogels, respectively. Integrating the bioactive dECM from differentiated cell sources allows GelO and GelC to induce differentiation in human adipose‐derived stem cells (hASCs) toward osteogenic and chondrogenic lineages. Further, GelO and GelC can be covalently adhered using a carbodiimide coupling reaction, forming a multi‐layered hydrogel with potential application as a bioactive osteochondral plug. The designed multi‐layered hydrogel can also induce differentiation of hASCs in vitro. In conclusion, the bioactive dECM carrying 3D printed robust hydrogel offers a promising new drug and cell‐free therapeutic strategy for bone and cartilage repair and future osteoarthritis management.

## Graphical abstract

Figure highlights the fabrication process of the osteogenic and chondrogenic decellularized extracellular matrix that can be used for repairing osteochondral defects. 3D osteochondral plugs containing GelMA and the bioactive decellularized extracellular matrix can be prepared by 3D printing.

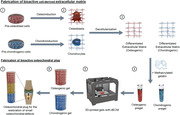

## 1 Introduction

Osteoarthritis (OA) and osteochondral defects (OCDs) are debilitating joint disorders characterized by progressive cartilage degradation and underlying bone abnormalities.^[^ ^1^, ^2^ ^]^ These conditions affect millions of individuals globally, leading to pain, loss of function, and reduced quality of life. Therefore, it is crucial to understand the prevalence of OA and OCDs and the limitations of current treatment modalities to explore novel therapeutic approaches. The current gold standard treatment options for OA and OCDs primarily aim to manage pain, relieve symptoms, or replace damaged tissue with transplantation and implantation.^[^ ^3^ ^]^ These approaches include grafting techniques, such as xenografts, allografts, and autografts. However, they pose several challenges, including a) immune rejection, b) the potential transmission of zoonotic diseases are major concerns associated with xenografts,^[^ ^4^ ^]^ c) variable long‐term outcomes,^[^ ^5^ ^]^ and d) limited availability of suitable autograft donor sites. As alternatives, synthetic biomimicking alloplastic materials, such as bioglass, hydroxyapatite, and collagen sponge, have been developed, showing promising therapeutic outcomes. However, challenges remain in achieving proper integration with native tissue, long‐term stability,^[^ ^6^ ^]^ natural remodeling capabilities,^[^ ^7^ ^]^ and limited control over structure.^[^ ^8^ ^]^

Recent advancements in therapies involving the use of cells, proteins, and peptides, either alone or in combination, have revolutionized the treatment of OA and OCDs. One of the most promising cell‐based therapies for osteochondral repair involves the use of stem cells.^[^ ^9^ ^]^ However, the heterogeneity and variability of cells obtained from different sources and donors present a significant limitation in stem cell‐based therapies for OA.^[^ ^10^ ^]^ In addition to cell‐based therapies, growth factors have emerged as essential components in regenerating damaged osteochondral tissue. Transforming growth factor‐beta (TGF‐β) and insulin‐like growth factor‐1 (IGF‐1) are two growth factors that have shown promising results in promoting cartilage formation and enhancing tissue healing.^[^ ^11^ ^]^ But aside from their high cost, there is a risk of off‐target effects and excessive tissue growth when growth factors are administered in high doses.^[^ ^12^ ^]^ Peptides have also gained attention as potential therapeutic agents for osteochondral repair but display poor stability^[^ ^13^ ^]^ and bioavailability. As a result, it is necessary to design therapeutic strategies that can act as effective alternatives to biobased drugs or cells.

Extracellular matrix (ECM)‐based therapeutics have become promising candidates for treating OA and OCDs. ECM can be harvested from various organs and tissues, which involves removing from living organisms or cadavers, followed by decellularization. In the field of osteochondral repair, where the regeneration of both cartilage and underlying bone is crucial, ECM extracted from cartilage and bone tissues has demonstrated significant potential.^[^ ^14^ ^]^ While tissue‐derived ECM offers several advantages in obtaining a native ECM composition and structure, it is not without limitations. One major drawback is the limited availability of donor organs or tissues, especially for specific applications.^[^ ^15^ ^]^ Besides availability, tissue‐derived ECM also suffers from high batch‐to‐batch variability. As a replacement, ECM‐derived from 2D tissue cultures has been proposed.^[^ ^16^ ^]^ By culturing cells in a controlled laboratory environment, cell‐derived ECM offers the advantage of scalability and consistency.

**Figure 1:**
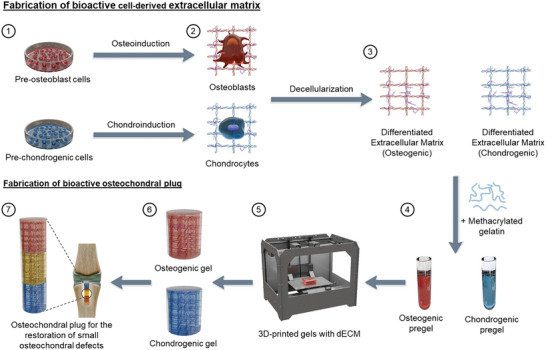
Illustration representing 3D printed robust multi‐layered hydrogel containing dECM for osteochondral repair. Pre‐osteoblast cells and pre‐chondrogenic cells were first cultured in a controlled laboratory environment (1). The cells were then differentiated toward osteogenic and chondrogenic lineages for 14 days (2). The deposited extracellular matrix was extracted by decellularization (3). The differentiated extracellular matrices were added to methacrylated gelatin to prepare osteogenic and chondrogenic pregels (4). The pregels were then 3D printed using a light‐based 3D printer. 5) Subsequently, gels capable of inducing osteogenesis and chondrogenesis in stem cells were prepared and characterized (6). Finally, the gels were adhered to using a carbodiimide coupling reaction to form multi‐layered hydrogel suitable for repairing small osteochondral defects.

Here, we have developed water‐swellable 3D, porous polymeric hydrogel scaffolds containing dECM from mammalian cell lines. As a critical first step for our proposed therapy, the cells were differentiated for fourteen days, allowing them to develop into a nutrition‐rich bioactive ECM. Subsequently, the culture was decellularized, and the extracted therapeutic dECM was added to the versatile biopolymer GelMA. GelMA is synthesized by methacrylating gelatin, which is derived from the naturally occurring structural protein, collagen.^[^ ^17^ ^]^ Being derived from natural sources, GelMA is highly biocompatible, and biodegradable, and contains arginine‐glycine‐aspartic acid (RGD) amino acid motifs that allow for cellular adhesion.^[^ ^18^, ^19^ ^]^ These properties of GelMA are similar to those of extracellular matrices, making them suitable candidates for osteochondral tissue repair. Furthermore, GelMA can undergo crosslinking via photoinitiated radical polymerization to form hydrogels.^[^ ^20^ ^]^ This method of crosslinking can be used to 3D print complex shapes using rapid, high‐resolution, and easy‐to‐use digital light processing (DLP)–based bioprinter.^[^ ^21^ ^]^ Here, the GelMA and dECM mixture were then 3D printed using a DLP bioprinter to obtain osteogenic, GelO, and chondrogenic, GelC scaffolds. These scaffolds could also be adhered to create multi‐layered plugs for potential osteochondral tissue repair. Such multi‐layered plugs aim to mimic the native tissue architecture of the articular cartilage, which consists of various layers.^[^ ^22^ ^]^ The chondrogenic layer is mechanically soft, whereas the osteogenic layer is comparatively stiffer. Although our present design establishes the validity of the approach by focussing on the bioactivity of the scaffolds, future designs will change the mechanical properties of the layers by changing GelMA concentrations, degree of methacrylation, photoinitiator concentration, time of photocrosslinking, and intensity of light exposure. We will also integrate nanomaterials that promote bone formation to mechanically reinforce the osteogenic layer. **Figure** **1** describes the steps involved in fabricating our 3D printed robust multi‐layered scaffold. Overall, our research contributes to advancing tissue engineering approaches for osteochondral tissue repair and brings us closer to providing effective treatments for patients suffering from OA and OCDs.

## 2 Results and Discussion

### 2.1 Bioactive dECM Can be Obtained from Differentiated MC3T3‐E1 and ATDC5 Cell Lines

Mouse calvaria‐derived preosteoblast cells, MC3T3‐E1 are widely used for investigating in vitro osteoblast differentiation and bone formation.^[^ ^23^, ^24^ ^]^ These cells proliferate rapidly, easily differentiate into osteoblasts, and secrete large quantities of ECM, making them the ideal cell source for this study. Similarly, ATDC5 is a mouse pre‐chondrogenic cell line derived from teratocarcinoma, which possesses characteristics of both chondrocytes (cartilage cells) and pre‐adipocytes.^[^ ^25^ ^]^ ATDC5 cells are widely used in research focused on cartilage development and chondrogenesis. Compared to other cells such as stem cells, the advantages of using these cell lines are but not limited to availability and ease of use, excessive ECM synthesis in a short time, excellent representation models, reproducibility, compatibility with molecular techniques, and standardized differentiation protocols.^[^ ^25^, ^26^, ^27^ ^]^

In this study, MC3T3‐E1 and ATDC5 cells were grown to confluency on 100 mm Petri dishes and differentiated using osteogenic differentiating media (ODM) and chondrogenic differentiating media (CDM), respectively, for 14 days. Supported by previous works,^[^ ^28^, ^29^ ^]^ dexamethasone served as the primary agent to induce osteogenesis in ODM, while for the preparation of CDM, a combination of human insulin, transferrin, and selenium (ITS) was used for chondroinduction. Furthermore, to enhance ECM deposition, L‐ascorbic acid and BGP were used in the preparation of the differentiating media. L‐ascorbic acid is a powerful antioxidant that plays a crucial role in the synthesis of collagen, a major component of the ECM, and enhances cell proliferation.^[^ ^30^ ^]^ BGP, on the other hand, is an organic phosphate compound that can induce mineralization in osteoblasts, and it has been shown to aid in the differentiation of osteoblasts and chondrocytes.^[^ ^31^, ^32^ ^]^ These two reagents are commonly used in culture systems aiming to study bone or cartilage development.

Differentiated osteoblastic and chondrogenic cell cultures were then treated with 1% Triton‐x100 to produce OdECM and CdECM. Triton‐X100 effectively solubilizes cell membranes, disrupting cellular structures and facilitating the removal of cellular contents with the preservation of the inherent bioactive molecules.^[^ ^33^ ^]^ The OdECM was scarped from the culture dish, washed, and then lyophilized to be used for further experiments. Each plate of MC3T3‐E1 produced 2.22 ± 0.40 mg of OdECM, and each plate of ATDC5 produced 2.80 ± 0.72 mg CdECM in their lyophilized form. Furthermore, the size distribution of dECM has been shown in Figures S1A,B (Supporting Information). Overall, this technique requires commonly accessible laboratory equipment and resources, making it convenient to implement within a typical laboratory environment.

### 2.2 Differentiated MC3T3‐E1 Cell‐Derived OdECM is Capable of Inducing Drug‐Free Osteogenesis in hASCs

**Figure 2:**
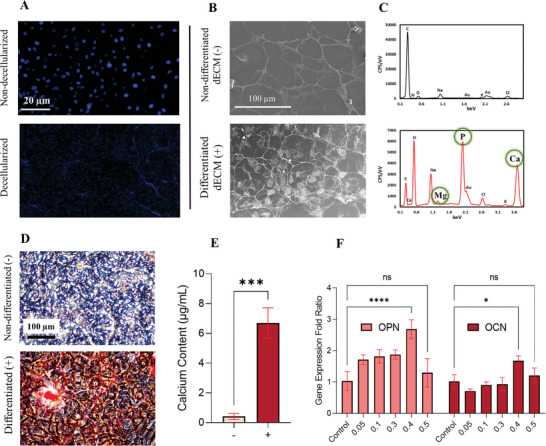
Bioactive OdECM from differentiated MC3T3‐E1 cells can induce drug‐free osteogenic differentiation in hASCs. A) DAPI staining of MC3T3‐E1 cells before (up) and after (down) decellularization, indicating successful removal of cellular material. B) Scanning Electron Microscopy (SEM) images comparing dECM derived from differentiated (+) and non‐differentiated (‐) MC3T3‐E1 cells, showing structural differences. C) Energy‐dispersive X‐ray (EDX) analysis of the two groups, revealing the relatively higher amount of minerals (Ca^2+^, Mg^2+^) in the differentiated dECM samples. D) Alizarin Red staining of the MC3T3‐E1 cells, confirming the mineralization potential of the differentiated dECM. E) Quantification of dye extraction from Alizarin Red staining, demonstrating the relative calcium levels in the groups (n=3). F) Reverse Transcription‐quantitative Polymerase Chain Reaction (RT‐qPCR) analysis of osteogenic markers (*OPN* and *OCN*) in human adipose‐derived stem cells (hASCs) treated with varying concentrations of OdECM (w/v), normalized to the *GAPDH* reference gene (n=3). 0.4% (w/v) concentration showed the highest gene expression (^*^ *p*<0.05, ^***^ *p*<0.001, ^****^ *p*<0.0001).

We first characterized and evaluated the OdECM extracted from differentiated MC3T3‐E1 cells. **Figure** **2**A demonstrates the successful removal of cellular material using DAPI staining, indicating the effectiveness of the decellularization process. This finding aligns with previous studies that have used Tritonx‐100 to remove cellular components from cell culture and generate acellular scaffolds.^[^ ^33^ ^]^ SEM images in Figure 2B reveal structural differences such as mineralization between dECM derived from differentiated (+) and non‐differentiated (‐) MC3T3‐E1 cells, respectively. These differences in dECM architecture may be attributed to the influence of cellular differentiation on the organization and composition of the dECM. Previous studies have reported that cell differentiation can alter the expression of ECM components, leading to variations in dECM structure.^[^ ^34^ ^]^ The energy dispersive x‐ray spectral chart in Figure 2C shows relatively higher amounts of mineral deposition such as calcium (Ca^2+^) and magnesium (Mg^2+^) in the OdECM samples. The mineralization potential of OdECM is particularly significant for bone tissue engineering applications, as the presence of Ca^2+^ is an indication of osteogenesis.^[^ ^35^ ^]^ The detection of minerals in the differentiated dECMs suggests that they possess the necessary components to support bone regeneration and is in line with previous studies that have reported successful mineralization of dECM derived from various cell types.^[^ ^36^ ^]^ The Alizarin Red staining in Figure 2D confirms the mineralization potential of the differentiated OdECM. The dECM(+) cells were positively stained compared to the control, indicating differentiation. As discussed, the deposition of calcium in the matrix indicates its ability to support mineralization processes. Figure 2E quantifies the relative calcium levels in the groups through dye extraction from ARS. ≈6 fold higher calcium levels in the differentiated dECM group suggest its superior mineralization capacity compared to the non‐differentiated group. The positive staining and higher calcium deposition in the differentiated dECM group further support its potential as a scaffold for bone tissue engineering.

In Figure 2F, RT‐qPCR analysis investigates the expression of osteogenic markers (*OPN* and *OCN*) in hASCs treated with varying concentrations of OdECM. *OPN* gene (also known as *SPP1*) encodes the protein osteopontin, which is involved in various functions related to bone remodeling, including cell adhesion, matrix mineralization, and regulation of osteoblast activity.^[^ ^37^ ^]^ It contributes to the regulation of bone formation, repair, and remodeling processes. *OCN* gene (also known as *BGLAP*) encodes the protein osteocalcin, which is a non‐collagenous protein found in bone and dentin.^[^ ^38^ ^]^ Osteocalcin plays a crucial role in regulating mineralization and the maturation of the ECM during bone formation. It is considered a marker of mature osteoblasts and is involved in the organization and deposition of hydroxyapatite crystals, contributing to bone mineralization.^[^ ^38^ ^]^ These genes are vital components of the complex regulatory network that governs osteogenesis and the maintenance of skeletal health.

From the RT‐qPCR analysis, it was found that a concentration of 0.4% (w/v) of OdECM leads to the highest expression of osteogenic markers. For this reason, a concentration of 0.4% was used for all other experimentations. This finding aligns with a related study that demonstrated successful bone formation in a mouse model using a PEG hydrogel incorporated with partially digested dECM derived from MC3T3‐E1 cells, with a concentration of 0.48% (w/v).^[^ ^39^ ^]^ To understand the underlying mechanism behind the expression of osteogenic markers (*OPN* and *OCN*), several factors come into play. OdECM likely provides a rich source of signalling molecules, such as growth factors, cytokines, and matrix proteins, which interact with cell surface receptors and trigger intracellular signalling pathways. One such pathway is the transforming growth factor‐beta (TGF‐β) signalling pathway, known to play a critical role in osteogenesis.^[^ ^40^ ^]^ TGF‐β stimulates the expression of osteogenic markers through the activation of downstream effectors such as Smads and Runx2.^[^ ^40^ ^]^ These effectors regulate the transcriptional activity of osteogenic genes, leading to enhanced expression of *OPN* and *OCN*. In addition to quantifying OPN and OCN upregulation, Figure S2 (Supporting Information) demonstrates the upregulation of RUNX2 when stem cells were differentiated using OdECM. These results provide valuable insights into the characterization of OdECM derived from differentiated pre‐osteoblastic cells. The successful decellularization, distinct structural differences, mineralization potential, and induction of osteogenic markers in hASCs support the suitability of differentiated dECM for bone tissue engineering applications.

### 2.3 Differentiated ATDC5 Cell‐Derived CdECM is Capable of Inducing Drug‐Free Chondrogenesis in hASCs

**Figure 3:**
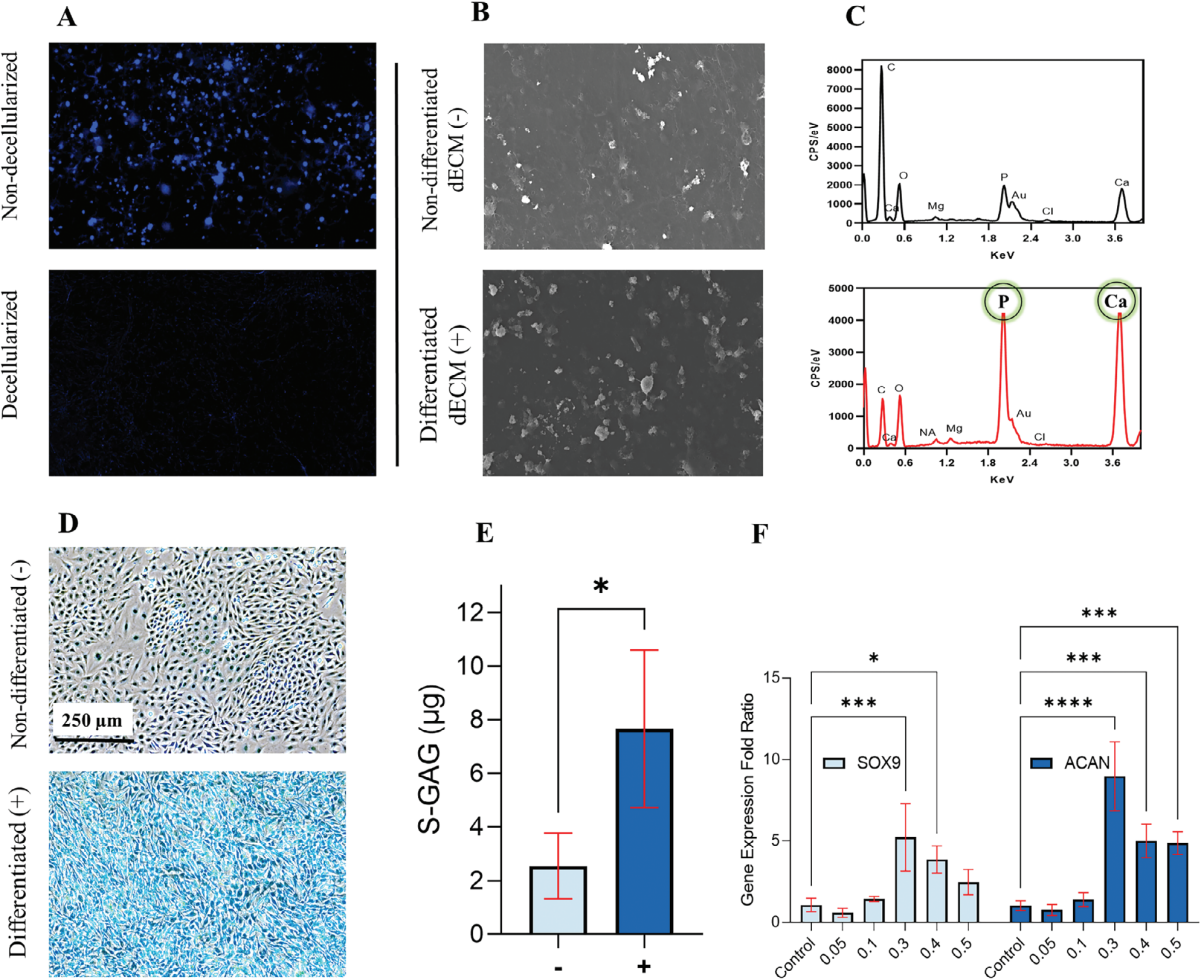
Bioactive CdECM from ATDC‐5 cells can induce drug‐free chondrogenic differentiation in hASCs. A) DAPI staining of ATDC5 cells before (up) and after (down) decellularization, indicating successful removal of cellular material. B) SEM images comparing dECM derived from differentiated (+) and non‐differentiated (‐) ATDC5 cells, showing structural differences. C) EDX analysis of the two groups, revealing the relatively higher amount of P and Ca+2 in the differentiated dECM samples. D) Alcian Blue staining of the ATDC5 cells, illustrating the presence of s‐GAG molecules in the differentiated dECM. E) Comparison of differentiated and non‐differentiated ATDC5 in s‐GAG synthesis (n=3). ATDC5 showed significantly higher synthesis of s‐GAGs upon introduction to chondrogenic differentiating media. F) RT‐qPCR analysis of chondrogenic markers (*SOX9* and *ACAN*) in hASCs treated with varying concentrations of CdECM (w/v), normalized to the GAPDH reference gene (n=3). 0.3% concentration showed the highest gene expression. (^*^ *p*<0.05, ^***^ *p*<0.001, ^****^ *p*<0.0001).

Next, we characterized and evaluated the CdECM extracted from differentiated ATDC5 cells, as shown in **Figure** **3**. DAPI images shown in Figure 3A capture the removal of DNA, suggesting successful decellularization. Figure 3B displays SEM images revealing the structural changes between dECM extracted from differentiated and non‐differentiated ATDC5 cells. The EDX spectra in Figure 3C demonstrate the increased production of inorganic components, including phosphates (P) and calcium (Ca^2+^) in the CdECM samples. Phosphate plays a crucial role in cartilage mineralization, cell differentiation, and apoptosis and has been implicated as a rate‐limiting factor in the process of cartilage repair.^[^ ^41^ ^]^ Through experiments using ATDC5 cells, it was found that phosphate promoted matrix mineralization and the expression of collagen X, a marker of chondrocyte maturation which highlights phosphate's regulatory role in chondrocyte maturation and apoptosis.^[^ ^42^ ^]^ Alcian Blue staining was performed on the ATDC5 cells to visualize the presence of sulfated glycosaminoglycan (s‐GAG) molecules in differentiated and non‐differentiated dECM (Figure 3D). A positively stained dECM(+) group indicates successful differentiation. Furthermore, s‐GAG quantification was performed between the two samples (Figure 3E). The staining demonstrated a robust and intense blue color, indicating the accumulation of s‐GAG molecules in the differentiated sample. The findings from s‐GAG quantification along with Alcian blue staining results suggest successful chondrogenic differentiation of the ATDC5 cells, as s‐GAGs are a hallmark of cartilage tissue and play a crucial role in maintaining its structure and function. The ability of the ATDC5 cells to differentiate into chondrocyte‐like cells and produce s‐GAGs has been well‐documented in various studies.^[^ ^25^, ^26^, ^43^ ^]^ Moreover, the significant increase in s‐GAG synthesis is in line with previous research that highlights the importance of insulin, transferrin, and selenium in driving chondrogenesis.^[^ ^26^ ^]^ The observed increase in s‐GAG synthesis can be attributed to the activation of various intracellular signalling pathways and transcription factors involved in chondrogenesis. These include *SOX9*, a master regulator of chondrogenic differentiation, which plays a crucial role in the synthesis of cartilage‐specific extracellular matrix components, including s‐GAGs.^[^ ^44^ ^]^ Differentiation likely upregulated the expression and activity of *SOX9*, leading to enhanced s‐GAG synthesis in the ATDC5 cells.

RT‐qPCR analysis was also conducted to investigate the expression of chondrogenic markers, specifically *SOX9* and *ACAN*, in hASCs treated with varying concentrations of CdECM derived from ATDC5 cells (Figure 3F). *SOX9* and *ACAN* are two critical genes involved in chondrogenesis, the process of cartilage formation and development.^[^ ^44^ ^]^ *SOX9* encodes a transcription factor that plays a central role in regulating the differentiation and maturation of chondrocytes. It is essential for the expression of numerous genes involved in cartilage development, including *ACAN*.^[^ ^44^ ^]^ *ACAN* encodes a large proteoglycan that constitutes a major component of ECM in cartilage tissue.^[^ ^45^ ^]^ It provides structural integrity to cartilage and is crucial for maintaining its compressive strength and hydration properties. The pathways involved in the regulation of *SOX9* and *ACAN* during chondrogenesis are complex and tightly coordinated. Several signalling pathways and transcription factors contribute to their expression and activity. One of the key pathways involved is the Wnt/β‐catenin signalling pathway.^[^ ^46^ ^]^ During the early stages of chondrogenesis, Wnt signalling is suppressed, allowing *SOX9* expression and subsequent chondrocyte differentiation.^[^ ^46^ ^]^ Later, as chondrocytes mature, Wnt signalling is activated, leading to the suppression of *SOX9* and the promotion of hypertrophic differentiation and enlargement.

The RT‐qPCR analysis revealed that a 0.3% concentration of CdECM resulted in the highest expression of chondrogenic markers in hASCs. This concentration was used for all future experiments. This finding indicates that the dECM derived from differentiated ATDC5 cells contains chondrogenic factors that enhance the chondrogenic potential of hASCs and influence the behavior of stem cells toward a chondrogenic phenotype.

### 2.4 Proteomics Analysis Further Establishes the Upregulation of Osteogenic and Chondrogenic Proteins Associated with OdECM and CdECM, Respectively

**Figure 4:**
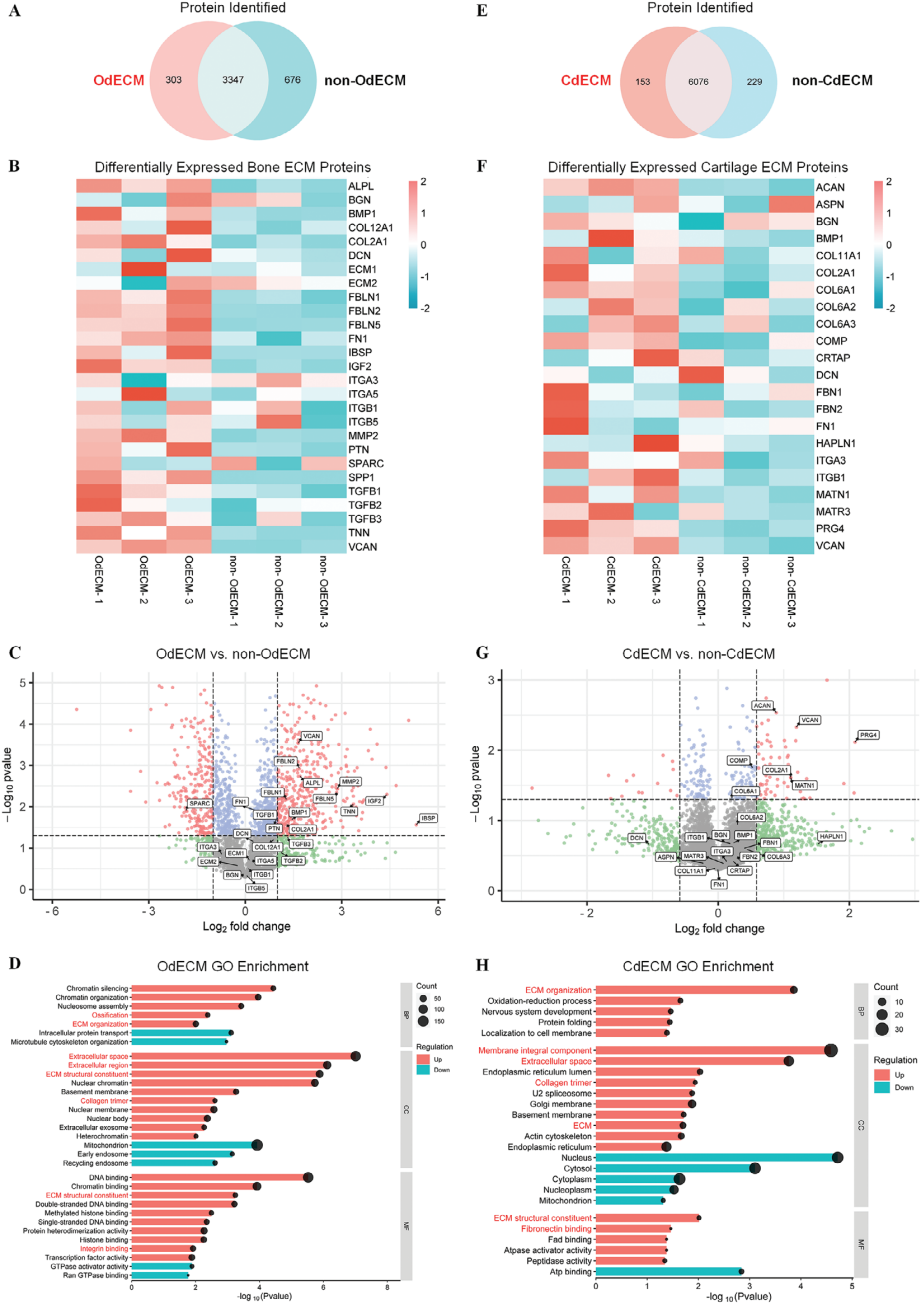
Proteomic Analysis of OdECM and CdECM. A) Venn diagram showing total protein identified in OdECM or non‐OdECM group. B) Heatmap of selected differentially expressed bone ECM proteins in OdECM and non‐OdECM. Red and blue represent up and downregulated expression, respectively. C) Volcano plot of differentially expressed proteins in OdECM relative to non‐OdECM. Typical bone‐related proteins were marked and shown in their gene name. D) Gene ontology enrichment analysis comparison between OdECM and non‐OdECM. Categories of the most differentially expressed gene sets with P < 0.05 are shown. E) Venn diagram showing total protein identified in CdECM or non‐CdECM group. F) Heatmap of selected differentially expressed cartilage ECM proteins in CdECM and non‐CdECM. Red and blue represent up and downregulated expression, respectively. G) Volcano plot of differentially expressed proteins in CdECM relative to non‐CdECM. Typical cartilage‐related proteins were marked and shown in their gene name H) Gene ontology enrichment analysis comparison between CdECM and non‐CdECM. Categories of the most differentially expressed gene sets with P < 0.01 are shown. N=3. GO, gene ontology; BP, biological process; CC, cellular component; MF, molecular function.

To further investigate the underlying molecular mechanism behind the osteogenic and chondrogenic ECM, proteomic analysis using high‐resolution MS technology was performed. The Venn diagram shows a total of 4326 proteins identified in Od/non‐OdECM samples with 303 proteins unique to OdECM (**Figure** **4**A). The variance of the samples has been presented in Figure S3 (Supporting Information). The heatmap displays an overall over‐expression of major bone ECM structure and signaling proteins in the OdECM group compared to the non‐OdECM group, demonstrating a more osteogenic ECM environment (Figure 4B).

Bone ECM mainly consists of hydroxyapatite as the primary mineral (70–90%), organic matrix (10–30%), and non‐collagenous proteins (≈10%).^[^ ^47^ ^]^ Type II collagen, consisting of α1 chain (COL2A1) and α2 chain (COL2A2), is the major type of structural protein in bone ECM (85–90%).^[^ ^48^ ^]^ Proteomic analysis showed COL2A1 upregulated to 1.4 fold with high significance (p = 0.02), indicating a more bone‐like ECM component in OdECM compared to non‐OdECM. Non‐collagenous proteins also play vital roles in promoting osteogenic differentiation, bone formation, and bone ECM signaling. As marked in the volcano plot, a large number of non‐collagenous bone ECM proteins were found to be significantly upregulated in OdECM compared to non‐OdECM (Figure 4C). These differentially expressed functional proteins include proteoglycans such as versican (VCAN, 3.1 fold) and decorin (DCN, 1.2 fold), and glycoproteins such as alkaline phosphatase (ALPL, 3.2 fold), osteopontin (SPP1, 128 fold), fibronectin (FN1, 1.3 fold) and bone sialoprotein (IBSP, 40 fold). Small bone ECM signaling proteins were also found to have significantly increased in OdECM compared to non‐OdECM, including insulin‐like growth factor II (IGF2, 21 fold), bone morphogenetic protein 1 (BMP1, 2.7 fold) and transforming growth factor beta‐1 (TGFB1, 1.8 fold). The Gene Ontology (GO) enrichment result showed significant up‐enrichment toward bone formation (ossification) in the biological processes, ECM and collagen in cellular components, and ECM structural constituent in molecular Functions (Figure 4D). Collectively, the protein profiles in OdECM displayed bone‐mimic ECM protein composition, which is consistent with the osteogenic results from in vitro experiments.

Figure 4E displays a Venn diagram showing 6458 proteins in Cd/non‐CdECM sample with 153 proteins unique to CdECM. This difference in proteomic profile compared to normal ECM indicates modified ECM environments of OdECM and CdECM. The overall expression of cartilage ECM proteins shown in the heatmap can be concluded to be a more chondrogenic ECM environment than non‐CdECM (Figure 4F). The CdECM proteomics analysis showed overexpression of cartilage major collagen components compared to non‐CdECM, including 1.1 fold increase in type II collagen (COL2A1, p = 0.02) and significant 0.2 fold increase in type VI collagen (Col6A1, p = 0.04) (Figure 4G)^[^ ^49^ ^]^ Interestingly, minor collagen components Type X collagen and Type XI were found to have no significant change. The other upregulated cartilage ECM non‐collagenous proteins include aggrecan (ACAN, 1.5 fold), cartilage oligomeric matrix protein (COMP, 1.4 fold), cartilage matrix protein (MATN1, 2.1 fold), proteoglycan 4 (PRG4, 4.2 fold). However, fibronectin (FN1) and cartilage‐associated protein (CRTAP) showed no significant differences in expression, probably because of sample size. The GO enrichment result showed significant up‐enrichment toward ECM‐related GO terms in biological process, cellular components, and molecular function with some direction toward cartilage ECM (Figure 4H). Still, the CdECM proteomics data together demonstrated enough cartilage promotive protein profile.

### 2.5 OdECM and CdECM Can be Integrated in GelMA Pregel and 3D Printed Using a Light‐Based 3D Printer

**Figure 5:**
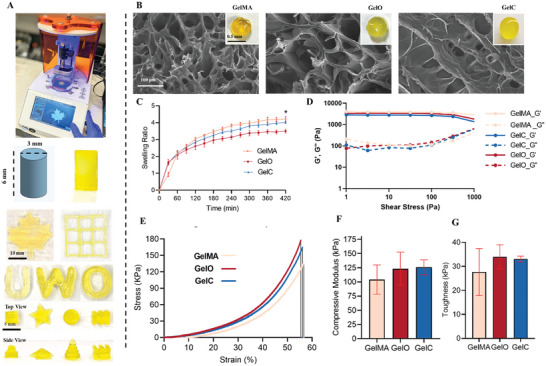
Bioactive OdECM and CdECM can be delivered using a 3D printable GelMA formulation. A) Schematic of DLP printer Lumen X programed with a cylindrical Onsahpe design and its corresponding GelC hydrogel for mechanical testing (diameter: 3mm, height: 6mm). Other hydrogels have been 3D‐printed to showcase the sensitivity of the printing technique. B) SEM images have been displayed revealing macroporous void spaces in the hydrogel. SEM visually showed the reduction in porosity upon the introduction of dECM. C) Graph exhibits the swelling profile of GelMA, GelO, and GelC (n=5). Swelling of GelO and GelC was reduced significantly compared to GelMA. D) Rheology experiment depicting the storage modulus (G') and loss modulus (G") profiles of GelMA, GelO, and GelC gels. Replicates (n=3) of 3 × 6 mm cylindrical gels were printed and hydrated in phosphate buffer saline for 5 min (representative replicate has been shown here). Stress sweep was performed at 37 °C from 0.1 to 104 Pa. The results demonstrate a very similar profile among the groups, with GelMA exhibiting a slightly higher storage modulus compared to GelO and GelC. E) A representative stress vs strain plot of formulated hydrogels obtained under uniaxial compression tests up to 0.6 mm mm^−1^ strain. F) Comparison between the compressive moduli of GelMA, GelO, and GelC hydrogels. An increase in compressive strength was observed with the incorporation of dECM. Compressive modulus was determined from the slope of the stress–strain plot in the region of 1–15% strain. Results are shown as mean ± standard deviation (n = 5). G) Toughness test results show a comparison of GelMA, GelO, and GelC gels. Toughness values (kPa) were measured to assess the resistance to fracture upon compressive force. The data reveals that the inclusion of dECM slightly increased the toughness in comparison to the samples without dECM (n=3). (* p<0.05).

Hydrogels containing OdECM (GelO) and CdECM (GelC) were printed using a Digital Light Processing (DLP)‐based printer. DLP printing uses light (often blue or UV light) to fabricate hydrogels with high precision and resolution. This technique works by projecting light patterns onto a liquid bioink, solidifying it layer by layer to form the desired 3D structure.^[^ ^50^ ^]^ The ability to create patient‐specific geometries is a significant advantage of 3D printing in tissue engineering. This customization allows for the fabrication of hydrogels that precisely match the defect or injury site, optimizing tissue regeneration outcomes.^[^ ^51^ ^]^ **Figure** **5**A shows the printer and the computer‐aided design used for fabricating the gels that were used for the mechanical characterization of the hydrogels. The corresponding hydrogels shown in the figure demonstrate the capability of the printing technique to produce precise and reproducible geometries, which is critical for fabricating tissue engineering scaffolds. Scanning Electron Microscopy (SEM) images were obtained to investigate the microstructure of the hydrogels (Figure 5B). The SEM analysis revealed macroporous void spaces in all the hydrogel formulations, indicative of their 3D network structure. The interconnected porous architecture of the hydrogels provides a favorable environment for cell infiltration, proliferation, and tissue regeneration. A reduction in porosity was evident upon the incorporation of dECM into the hydrogels. This finding suggests that the incorporation of dECM leads to a more densely crosslinked network, potentially enhancing the overall mechanical properties of the hydrogels. The increased crosslinking density with dECM might be attributed to enhanced interaction between dECM particles and the polymer network of GelMA due to the presence of additional functional groups and reactive sites. Additionally, it is plausible that the introduction of dECM reduced the penetrance of blue light during the photo‐crosslinking process, resulting in a tighter and less porous network formation.

### 2.6 Incorporation of OdECM and CdECM Reduces Swelling and Degradation of the GelMA Hydrogels

The swelling profiles of hydrogels were investigated to understand their water uptake capacities (Figure 5C). The ability of hydrogels to absorb and retain water is essential for maintaining a hydrated environment within the scaffold, supporting cell growth, and facilitating nutrient diffusion. The results demonstrate that GelO and GelC exhibited significantly reduced swelling compared to GelMA. The reduction in swelling indicates a tighter network structure in GelO and GelC, corroborating the SEM observations. The diminished swelling behavior may be attributed to the presence of dECM, which could restrict water penetration and result in a more compact hydrogel structure. Additionally, the dECM molecules may sterically hinder the access of water molecules to the hydrogel matrix, leading to reduced swelling. We also investigated the degradation behavior of the hydrogels on day 10. Degradation studies provide valuable insights into the hydrogel's ability to maintain structural integrity and its susceptibility to breakdown over time. The percentage of degradation was measured as 2.012 ± 0.38 for GelMA, 1.69 ± 0.10 for GelO, and 1.87 ± 0.38 for GelC. The results indicated that GelO and GelC hydrogels exhibited relatively lower degradation rates compared to GelMA. This reduction in degradation may be attributed to the incorporation of dECM, which could act as a protective barrier. Similar to swelling results, the presence of dECM particles may also create additional crosslinks, further stabilizing the hydrogel structure and contributing to its enhanced resistance against degradation.

### 2.7 OdECM and CdECM Maintain the Rheological Properties of the GelMA Hydrogels

Rheological experiments were conducted to assess the viscoelastic properties of GelMA, GelO, and GelC hydrogels (Figure 5D). The storage modulus (G“) and loss modulus (G”) profiles were measured by subjecting the cylindrical gels to a stress sweep at 37 °C. The rheological behavior of hydrogels is crucial as it reflects their mechanical stability, ability to withstand external forces, and their potential as tissue engineering scaffolds. Remarkably, the three hydrogel formulations exhibited very similar rheological profiles. GelMA displayed a slightly higher storage modulus compared to GelO and GelC, suggesting that it possesses greater elastic behavior and resistance to deformation. The slight variation in viscoelastic behavior may arise from differences in the molecular weight and density of the hydrogel networks.

### 2.8 OdECM and CdECM Enhance the Mechanical Strength of GelMA Hydrogels

Uniaxial compression tests were conducted to analyze the compressive strength of the formulated hydrogels (Figure 5E). The stress versus strain plots demonstrated the mechanical response of the hydrogels under compressive force up to 0.6 mm mm^−1^ strain. The compressive strength of hydrogels is essential in supporting and maintaining tissue structure under mechanical loading. Figure 5F presents a comparison of the compressive moduli of GelMA, GelO, and GelC hydrogels within the linear section of the stress–strain curve. The data revealed an increase in compressive strength with the incorporation of dECM. GelO and GelC hydrogels, with dECM, exhibited enhanced mechanical stiffness compared to GelMA, suggesting that the presence of dECM contributes to the overall reinforcement of the hydrogel matrix. The integration of dECM potentially creates a composite‐like structure, combining the mechanical properties of both components and resulting in a stiffer and more robust hydrogel.

Next, toughness was determined to assess the ability of the hydrogels to resist fracture upon being subjected to compressive force (Figure 5G). The results showed that the inclusion of both OdECM and CdECM slightly increased the toughness of GelO and GelC hydrogels compared to their counterparts without dECM. Toughness is a critical mechanical property in tissue engineering as it reflects the energy required to induce failure in the hydrogel. The improved toughness can be attributed to structural integrity conferred by the presence of dECM, which enhances the hydrogel's resistance to fracture and deformation. The improved toughness indicates that the hydrogels with dECM can be desirable candidates for non‐load‐bearing applications.

The observed improvements in mechanical properties, coupled with the ability to control structural characteristics through 3D printing, underscore the potential of GelO and GelC hydrogels as promising biomaterials for osteochondral repair applications. Continued investigation into the effects of dECM concentration and other modifications on hydrogel properties will help refine and tailor these hydrogels for specific bone and cartilage tissue engineering applications.

### 2.9 Cytocompatible GelO Can Induce Drug and Cell‐Free Osteogenic Differentiation in hASCs

After establishing a formulation for Gel, we aimed to evaluate the cytocompatibility of the bioactive hydrogel by investigating the organization and morphology of cytoskeleton and actin filaments, as well as cell viability and metabolic activity (Figure S4A, Supporting Information). Fluorescent staining using Phalloidin (green) and DAPI (blue) was employed to assess the structural integrity of hASCs cultured on thin layers of GelMA and GelO for 24 h.

The staining results revealed that GelMA and GelO hydrogels supported the preservation of the organization and morphology of the cytoskeleton and actin filaments, as indicated by the intense green fluorescence. This suggests that the hydrogels did not disrupt the structural integrity of hASCs, demonstrating their cytocompatibility. Additionally, the blue fluorescence from DAPI staining indicated that the cell nuclei remained intact, further reducing the possibility of genotoxic effects induced by the hydrogels. The maintenance of cellular architecture is of utmost importance, as it directly influences cell behavior, including adhesion, migration, and differentiation. To assess the metabolic activity and cell viability of hASCs cultured on GelO, an MTS assay was conducted, as displayed in the bar graph (Figure S4A, Supporting Information). The data indicated that both GelMA and GelO hydrogels were able to support the survival and proliferation of hASCs, as evidenced by the similar metabolic activity observed in both groups. GelMA hydrogels have been recognized for their potential in wound healing, drug delivery, biosensing, and tissue regeneration due to their biocompatibility and tunable physical properties.^[^ ^52^ ^]^ Our findings showed the addition of dECM into GelMA retained the cytocompatibility of hydrogels. This suggests that these hydrogels can serve as suitable substrates for supporting osteogenic differentiation.

After demonstrating cytocompatibility, we evaluated the osteogenic potential of the developed GelO. Figure S4B (Supporting Information) illustrates the corresponding experimental setup, where Alizarin red stain was used to determine the deposition of minerals. The hASCs were cultured on a thin layer of hydrogels for 21 days to induce osteogenic differentiation. Micrographs of the Alizarin Red stain have been displayed in Figure S4C (Supporting Information). Here, the positive group designated as OI was treated with osteoinductive media containing dexamethasone in DMEM. OI served as a positive control that induced osteogenic differentiation with the help of drugs. We chose to use DMEM as basal media for positive control because it has been reported that stem cells show a maximal differentiation in DMEM supplemented with appropriate drugs or serums compared to other types of media.^[^ ^27^, ^53^, ^54^, ^55^ ^]^ Intense red in OI was observed due to the induction of differentiation by dexamethasone. The other groups were cultured in hASC basal media without the osteoinductive factors. It can be observed that the hASCs grown on GelO exhibited visible mineralization, indicating successful osteogenic differentiation (Figure S4C, Supporting Information). On the other hand, the GelO (‐) group, which refers to the hydrogel containing 0.4% (w/v) dECM derived from non‐differentiated MC3T3‐E1 cells, showed similar color intensity to the GelMA control group. This finding suggests that differentiation of pre‐osteoblastic cells is essential to harvest osteoinductive dECM. The quantitative analysis of the Alizarin Red staining results is depicted in Figure S4D (Supporting Information). The staining levels were measured by extracting the dye and quantifying its absorbance at 405 nm. The results demonstrated that hASCs treated with GelO hydrogel exhibited significantly higher levels of staining compared to the control groups, indicating enhanced osteogenic differentiation. The GelO hydrogel likely provides a conducive microenvironment for hASCs, facilitating their commitment toward the osteogenic lineage.

### 2.10 Cytocompatible GelC Can Induce Drug‐Free Chondrogenic Differentiation in hASCs

Figure S5A (Supporting Information) demonstrates the cytocompatibility of GelC using hASCs. Phalloidin and DAPI stains demonstrate that GelC does not disrupt the structural integrity of hASCs grown on the hydrogels. Further, the MTS assay indicates that GelC supports cell survival and growth. Next, the chondrogenic potential of GelC was determined. Here too, the cells were cultured on a layer of GelC for 21 days. The cells were then stained with Alcian blue, as shown in the illustration for the experimental setup (Figure S5B, Supporting Information). A chondroinductive media containing DMEM/F12 was used as a positive control (CI). An intense blue color was observed in the positive control because of the inevitable differentiation from the use of commercial chondroinductive drugs. The hASCs cultured on GelC hydrogel showed a prominent and intense blue coloration, indicative of higher production of sulfated glycosaminoglycans (s‐GAGs). This robust s‐GAG production observed in the GelC group provides strong evidence of successful chondrogenic differentiation, implying the formation of cartilage‐like structures (Figure S5C, Supporting Information). In contrast, the GelC (‐) group, which consisted of the hydrogel containing 0.3% (w/v) dECM derived from non‐differentiated ATDC5 cells, displayed a color intensity comparable to that of the GelMA control group. This finding suggests that the differentiation of pre‐chondrogenic cells is crucial for obtaining chondroinductive dECM, as the GelC (‐) group lacks the distinct blue coloration associated with increased s‐GAG production.

Figure S5D (Supporting Information) presents the quantitative analysis derived from the Sulfated‐Glycosaminoglycans Assay Kit. This assay efficiently detects all s‐GAGs, including heparan sulfate, chondroitin sulfate, and keratan sulfate. The principle of the assay involves the interaction of a cationic dye, 1,9‐dimethylmethlene Blue (DMMB), with the highly negatively charged s‐GAGs, resulting in the formation of a colored product. The absorbance of this colored signal is measured at 525 nm and is directly proportional to the concentration of sulfated GAGs present in the sample. The outcome of the quantitative analysis clearly indicated that hASCs treated with the GelC hydrogel exhibited significantly higher levels of staining compared to the control groups. This observation strongly suggests enhanced chondrogenic differentiation in the GelC group. The GelC hydrogel likely provides a favorable microenvironment that promotes hASCs' commitment toward the chondrogenic lineage, leading to the augmented production of sulfated GAGs.

### 2.11 Combined GelO and GelC Can Simultaneously Induce Osteogenesis and Chondrogenesis in hASCs

**Figure 6:**
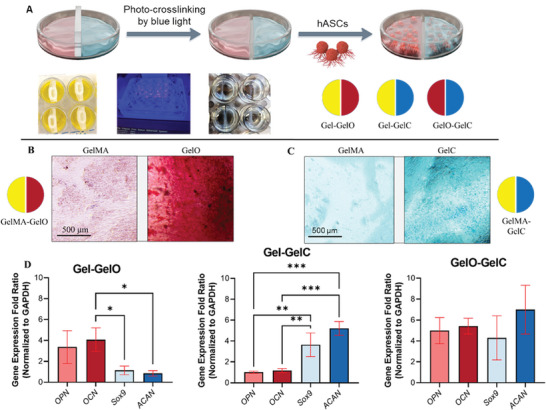
A combined GelO and GelC can simultaneously promote osteogenic and chondrogenic differentiation in hASCs. A) The illustration depicts the experimental design for laterally‐placed thin layer hydrogel treatments in a 24‐well plate. A plastic tight wall was inserted in the middle of the wells, creating separate compartments for GelMA, GelO, and GelC. The blue light was applied for photo‐crosslinking of the hydrogel premixes. hASCs were seeded on top of the hydrogels and cultured for 21 days to analyze gene expression. B) Alizarin Red staining was performed on Gel‐GelO samples to assess osteogenic differentiation. The bright red color observed on the GelO side indicated the presence of calcium deposits, indicative of successful osteogenesis. In contrast, the GelMA side exhibited limited red staining, suggesting minimal osteogenic differentiation. C) Alcian Blue staining was conducted on Gel‐GelC samples to evaluate chondrogenic differentiation. The presence of a bright blue color on the GelC side indicated the deposition of sulfated proteoglycans, a characteristic feature of chondrogenesis. Conversely, the GelMA side showed minimal blue staining, indicating limited chondrogenic differentiation. D) RT‐qPCR analysis was performed to quantify the expression levels of osteogenic markers (OPN and OCN) and chondrogenic markers (SOX9 and ACAN) in hASCs treated with Gel‐GelO, Gel‐GelC, and GelO‐GelC. The gene expression levels were normalized to the reference gene GAPDH. The Gel‐GelO group exhibited significantly higher expression levels of osteogenic markers, indicating enhanced osteogenic differentiation compared to the other groups. Similarly, the Gel‐GelC group demonstrated higher expression levels of chondrogenic markers, suggesting enhanced chondrogenic differentiation. Notably, the GelO‐GelC group exhibited a remarkable 4 to 6‐fold upregulation of both osteogenic and chondrogenic markers, indicating synergistic effects of GelO and GelC in promoting both lineages (^*^ *p*<0.05, ^**^ *p*<0.01, ^***^ *p*<0.001).

Osteogenesis and chondrogenesis are vital processes in osteochondral tissue repair. The ability to promote the differentiation of stem cells into osteoblasts and chondrocytes using hydrogel‐based treatments is of great interest and is the final goal of this study. Here, we sought to evaluate the effectiveness of three different hydrogel combinations (Gel‐GelO, Gel‐GelC, GelO‐GelC) in inducing osteogenic and chondrogenic differentiation (**Figure** **6**). The experimental design involved a lateral deposition of thin‐layer hydrogel treatments, and gene expression analysis was used to quantify the differentiation outcomes.

The setup included a 24‐well plate with separate compartments created by a plastic tight wall inserted in the middle (as shown in Figure 6A). The three hydrogel types used were GelMA, GelO, and GelC. The blue light was applied to photo‐crosslink the hydrogel premixes. hASCs were seeded on top of the hydrogels and cultured for 21 days to allow for osteogenic and chondrogenic differentiation. For the assessment of osteogenesis, Alizarin Red staining was performed on Gel‐GelO samples (Figure 6B). The GelO side displayed a bright red color, indicating the presence of calcium deposits. In contrast, the GelMA side exhibited limited red staining, suggesting minimal osteogenic differentiation. This result indicates that GelO was more effective than GelMA in promoting the osteogenic differentiation of hASCs. To evaluate chondrogenesis, Alcian Blue staining was conducted on Gel‐GelC samples (Figure 6C). The GelC side displayed a bright blue color, indicating the deposition of sulfated proteoglycans, a characteristic feature of chondrogenesis. On the other hand, the GelMA side exhibited limited blue staining, suggesting limited chondrogenic differentiation. This finding suggests that GelC was more effective in promoting chondrogenic differentiation compared to GelMA within the same well.

To quantify the expression levels of osteogenic and chondrogenic markers, RT‐qPCR analysis was performed on hASCs treated with different hydrogel combinations (Figure 6D). The gene expression levels were normalized to the reference gene *GAPDH*. The Gel‐GelO group showed significantly higher expression levels of osteogenic markers, *OPN*, and *OCN*, indicating enhanced osteogenic differentiation compared to the other groups. Furthermore, the Gel‐GelC group demonstrated higher expression levels of chondrogenic markers, *SOX9*, and *ACAN*, suggesting enhanced chondrogenic differentiation compared to the other groups. These results are consistent with the findings from Alizarin Red and Alcian Blue staining, indicating that GelO and GelC effectively promote lineage‐specific differentiation in hASCs.

The most intriguing finding of this study was observed in the GelO‐GelC group. This group exhibited a remarkable 4 to 6‐fold upregulation of both osteogenic and chondrogenic markers, indicating synergistic effects of GelO and GelC in promoting both lineages. The increased expression of *OPN* and *OCN* suggests that the combination of GelO and GelC not only enhances osteogenesis but also provides an osteoinductive microenvironment promoting the commitment of hASCs toward osteoblast‐like cells. Similarly, the upregulation of chondrogenic markers, indicates that the combination of GelO and GelC synergistically induces chondrogenic differentiation in hASCs. The observed synergistic effect of GelO and GelC can be attributed to their distinct mechanisms of action. During early osteogenesis, cells are known to release osteoinductive factors such as Runx2 that stimulate the differentiation of hASCs into osteoblasts.^[^ ^56^ ^]^ Runx2 is one of the earliest transcription factors upregulated during osteoblast differentiation, and its expression is essential for the commitment of MSCs to the osteoblastic lineage. Runx2 acts as a transcriptional activator, binding to specific DNA sequences in the regulatory regions of numerous osteogenic genes including *OCN* and *OPN* to stimulate their expression.^[^ ^56^ ^]^ As osteoblasts mature Runx2 levels decrease, and this fluctuation also affects chondrogenesis. In the early stages of chondrogenic differentiation, *Runx2* expression is upregulated in stem cells as they condense to form chondroprogenitor cells.^[^ ^57^ ^]^ *Runx2* is also involved in the transition of chondrocytes to hypertrophic chondrocytes, a process that occurs during endochondral ossification.^[^ ^57^ ^]^ In this process, chondrocytes in the center of the cartilage model enlarge and undergo mineralization, leading to the formation of the primary ossification center. *Runx2* expression is upregulated in hypertrophic chondrocytes and plays a role in the mineralization of the cartilage matrix.^[^ ^57^ ^]^ However, as chondrogenesis progresses, *Runx2* expression is downregulated in committed chondrocytes. Therefore, the combination of GelO and GelC hydrogels likely creates a multifunctional microenvironment that enhances the expression of lineage‐specific transcription factors, promoting both osteogenesis and chondrogenesis. The observed synergistic effect of GelO and GelC in promoting both osteogenesis and chondrogenesis aligns with the notion that combining multiple cues can lead to enhanced differentiation outcomes. Previous studies have shown that biomaterials can act synergistically to regulate stem cell fate. For example, researchers fabricated a biphasic hydrogel called CAN‐PAC to facilitate the regeneration of osteochondral defects.^[^ ^58^ ^]^ In an experimental rabbit model, bilayer hydrogels were applied to the defect sites. The regenerated tissues exhibited newly formed transparent cartilage and repaired subchondral bone, providing evidence of the hydrogel's efficacy in promoting osteochondral defect repair.^[^ ^58^ ^]^

This comparative analysis of thin‐layer hydrogel treatments demonstrated the differential abilities of GelO and GelC in promoting osteogenesis and chondrogenesis. The GelO‐GelC combination displayed a remarkable synergistic effect in promoting both lineages, making it a promising candidate for future osteochondral repair applications. Further investigations are warranted to understand the underlying mechanisms responsible for this synergistic effect and to optimize the hydrogel composition and culture conditions.

### 2.12 Covalently Adhering GelO and GelC to form a Bioactive Multi‐Layered Hydrogel Plug with the Potential to Repair Small Osteochondral Defects

**Figure 7:**
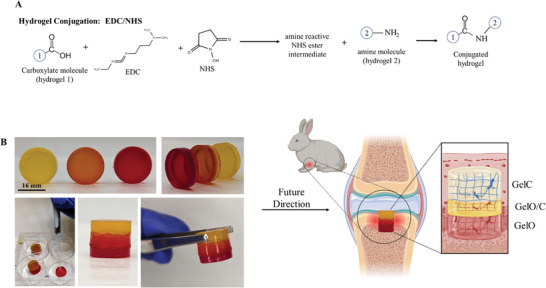
GelO and GelC can be conjugated to form a multi‐layered hydrogel using an EDC/NHS coupling reaction. A) The figure provides an illustration depicting the process of conjugating GelMA hydrogels. EDC (1‐Ethyl‐3‐(3‐dimethylaminopropyl) carbodiimide) and NHS (N‐Hydroxysuccinimide) are used as coupling agents to covalently link the GelMA hydrogels together. This conjugation process facilitates the creation of a structurally stable and functional three‐layer hydrogel. B) The figure presents a conceptual design for an osteochondral plug that can be potentially utilized in future applications. The osteochondral plug is composed of three distinct layers: a chondrogenic layer (GelC) to promote cartilage formation, an interphase layer (GelO/C) to facilitate the integration between cartilage and bone, and an osteogenic layer (GelO) to support bone regeneration. The GelMA hydrogels in each layer are conjugated through EDC/NHS coupling, ensuring structural integrity and compatibility. By implanting the three‐layer hydrogel into the osteochondral defect site, the plug aims to facilitate the regeneration of both cartilage and bone, promoting functional tissue repair.

The process of conjugating GelMA hydrogels through EDC/NHS coupling for multi‐layered hydrogel design offers a promising approach to osteochondral tissue repair. In **Figure** **7**A, a detailed illustration showcases the step‐by‐step process of conjugating two GelMA hydrogels through EDC/NHS coupling. EDC acts as a crosslinker that activates carboxylic acid groups on the GelMA hydrogels, generating highly reactive O‐acylisourea intermediates.^[^ ^59^ ^]^ NHS, on the other hand, functions as a stabilizing agent for the O‐acylisourea intermediates. The O‐acylisourea intermediate readily reacts with primary amines present on the second GelMA chains, resulting in amide bond formation, and effectively conjugates the GelMA hydrogels.^[^ ^59^ ^]^ The use of EDC/NHS coupling provides several advantages in adhering multiple layers together and has been used frequently for hydrogel fabrication and tissue adherence.^[^ ^60^, ^61^ ^]^ First, the process ensures a strong and stable covalent linkage between the hydrogels, enhancing the structural integrity of the composite material.^[^ ^62^ ^]^ This is critical for its successful implantation and long‐term performance in the challenging osteochondral defect environment. Second, the covalent bonds formed via EDC/NHS coupling do not adversely affect the biocompatibility of the hydrogel as studies have used this technique in in vitro and in vivo studies.^[^ ^63^, ^64^, ^65^ ^]^

The conceptual design of the multi‐layered hydrogel plug, consisting of three distinct layers, showcases the versatility of the EDC/NHS coupled GelMA hydrogels with possible future applications in osteochondral tissue repair (Figure 7B). The first layer, the chondrogenic layer (GelC), specifically aims to stimulate cartilage formation. The second layer, the interphase layer (GelO/C), serves as a transitional zone between the cartilage and bone layers, which can facilitate a smooth and continuous surface for load transmission and joint function. The third layer, the osteogenic layer (GelO), focuses on promoting bone regeneration by providing a conducive environment for stem cell attachment and differentiation. By integrating the multi‐layered hydrogel into the osteochondral defect site, the developed osteochondral plug may have a promising effect on the regeneration of both cartilage and bone. Future investigations are needed to explore the mechanical properties, cell adhesion, overall biocompatibility, and differentiation potential of the designed multi‐layered hydrogel.

## 3 Conclusion

In this study, we developed a bioactive gelatin‐based hydrogel scaffold for application in osteochondral tissue repair, combining dECM isolated from differentiated bone and cartilage cell lines. While animal tissue‐derived dECM suffers from the variability due to age, gender, and underlying health conditions,^[^ ^66^ ^]^ 2D tissue culture‐derived dECM avoids these issues, can be abundantly manufactured, and is free from pathogen. Further, an optimized dECM production in a lab‐controlled environment can provide high batch‐to‐batch consistency.^[^ ^67^, ^68^ ^]^ For the decellularization process, we used Triton X‐100, but there are other available strategies, such as using sodium deoxycholate, sodium dodecyl sulfate, repeated freeze/thaw cycles, or TritonX‐100 in combination with DNase, that can be explored in future for effective cellular component removal.^[^ ^69^ ^]^ However, care must be taken in maintaining the bioactivity of the dECM when using any decellularization protocol to maximize the therapeutic outcome. The results of our experiments demonstrate the potential of this dECM‐containing hydrogel as a drug and cell‐free treatment option for OA and OCDs, simultaneously regenerating the articular cartilage and subchondral bone region of the osteochondral interface. The production and characterization of dECM from MC3T3‐E1 and ATDC5 cell lines proved to be efficient and reproducible, yielding significant amounts of decellularized matrices with preserved bioactive molecules. SEM imaging and staining techniques confirmed the successful decellularization and provided insights into the structural and mineralization differences between differentiated and non‐differentiated dECM. In addition, the quantitative analysis of calcium levels and sulfated glycosaminoglycans in the dECM supported their osteogenic and chondrogenic properties, respectively. Furthermore, the quantitative proteomics further identified the upregulation of bone ECM component proteins in OdECM and cartilage ECM component proteins in CdECM compared to their control group, respectively. In‐depth bioinformatic analysis ultimately revealed the bone‐mimic protein profile toward osteogenesis in OdECM and the cartilage‐mimic protein profile toward chondrogenesis in CdECM at the molecular biology level.

The physicochemical and mechanical characterization of GelO and GelC hydrogels revealed improved mechanical stiffness and reduced swelling and degradation profiles compared to the GelMA control group. These findings indicate that the incorporation of dECM positively influences the hydrogel's mechanical properties, making it a highly suitable candidate for osteochondral repair applications. It should also be noted that the mechanical strength of osteochondral plugs varies between layers. As such, it is crucial to also focus on optimizing material composition based on the tissue type. In our future studies, GelMA in the osteogenic layer will be mechanically strengthened using standard reinforcement strategies, such as integrating nanoparticles or polymers.^[^ ^70^ ^]^ Softer scaffolds containing only GelMA will be used for the chondrogenic layer. Additionally, cytocompatibility studies demonstrated that both GelO and GelC hydrogels supported the survival, proliferation, and differentiation of hASCs toward osteogenic and chondrogenic lineages. Alizarin Red and Alcian Blue staining confirmed the osteogenic and chondrogenic potential of GelO and GelC, respectively. Interestingly, the combination of GelO and GelC in a multi‐layered configuration displayed a remarkable synergistic effect in promoting both osteogenesis and chondrogenesis, making it a promising candidate for osteochondral repair. Finally, the covalent conjugation of GelMA hydrogels using carbodiimide coupling chemistry allows for the design of a multi‐layered plug for the osteochondral tissue layer.

While this study has provided significant insights into developing and characterizing the designed multi‐layered bioactive hydrogel plug, further investigations are warranted to explore its long‐term performance, in vivo biocompatibility, and regenerative properties. Moreover, scale‐up will be limited primarily by dECM production. Current manufacturing protocols are optimized for cell extraction, and not for the procurement of dECM.^[^ ^71^, ^72^, ^73^ ^]^ Therefore, proper scale‐up, with high product consistency, will require substantial process parameter optimization. As such, care must be taken in maintaining the tissue culture and observing the senescence of the cultured cells. Extraction protocols must be standardized, and rigorous quality control measures should be undertaken to warrant superior therapeutic efficacy. Automation will further reduce batch‐to‐batch variability and ensure high throughput production. Overall, we have developed an advanced bioactive 3D printable multi‐layered hydrogel plug for promoting osteochondral tissue repair, eliminating the need to incorporate drugs or cells. Our simplistic material design and easy‐to‐fabricate strategies hold tremendous potential in treating OA and OCDs.

## 4 Experimental Section

### dECM Extraction from Pre‐Osteoblastic Murine Cells (MC3T3‐E1)

Pre‐osteoblastic MC3T3‐E1 cell line (ATCC, CRL‐2593, subclone 4) were grown in an expansion medium which contained α‐Minimum Essential powdered Medium (α‐MEM; Sigma, M0644) dissolved in autoclaved DI water and supplemented with 10% Fetal Bovine Serum (FBS; Sigma, F1051) and 1% PenStrep (PS; Gibco, 15070‐63). For passaging, cells were treated with 0.25% Trypsin‐EDTA (Gibco, 25200072) for 10 min in an incubator (Thermo Forma Steri Cycle 370, US). Passaging was performed at 90% cellular confluency. To induce osteogenesis in the MC3T3‐E1 cell monolayer, 5 mL of Osteogenic Differentiating Media (ODM) was added when cells reached confluency with a total change of media every 3 days. ODM was made using the basal medium supplemented with 10 mM β‐glycerophosphate disodium salt hydrate (BGP, sigma, G9422) and100 nM Dexamethasone (Dex, Sigma, D4902). For ECM production, 1.5 × 10^6^ Cells were seeded onto 100 mm Petri dishes and incubated at 37 °C with 5% CO_2_. After 14 days of differentiation without passaging, the ECM monolayer was treated with 1% Triton X‐100 in PBS (v/v) with 1% PS for 10 min, following standard decellularization protocols with minor modifications.^[^ ^74^, ^75^, ^76^ ^]^ The differentiated dECM monolayer (OdECM) was washed 4 times with 5 mL of PBS with 1% PS and then washed in 5 mL of water overnight at 4 °C. After the removal of excessive water, the monolayer was almost airdried in a biosafety cabinet. Damp dECM was scraped from the petri dish, lyophilized, and stored at −80 °C until use.

### dECM Extraction from Pre‐Chondrogenic Murine Cells (ATDC5)

The pre‐chondrogenic ATDC5 cell line was a gift from Dr. Frank Beier (Western University, Canada). The cells were cultured in expansion medium Dulbecco's modified Eagle's medium with nutrient mixture F‐12 (DMEM/F12; Thermo Fisher, 11320033) and supplemented with 5% FBS, 1% PS, 10 mM BGP, and 0.5 mM L‐ascorbic acid (Sigma, A4544). For passaging, cells were trypsinized for 5 min in the incubator. To induce chondrogenesis in the ATDC5 cell monolayer, 5 mL of Chondrogenic Differentiating Media (CDM) was used with a total change of media every 3 days. CDM was made using the expansion medium supplemented with 1% ITS 100X (Sigma, I3146). For ECM production, 1.5 × 10^6^ Cells were seeded onto 100 mm Petri dishes and incubated at 37 °C with 5% CO_2_. After 14 days of differentiation without cell splitting, the ECM monolayer was treated with 1% Triton X‐100 in PBS (v/v) with 1% PS for 10 min. The dECM monolayer (CdECM) was washed 4 times with 5 mL of PBS with 1% PS and then washed in 5 mL of water overnight at 4 °C. After the removal of excessive water, the monolayer was almost airdried in a biosafety cabinet. Damp dECM was scrapped from the petri dish, lyophilized, and stored at −80 °C until use.

### In Vitro Feasibility Analysis Using Human Adipose‐Derived Stem Cells (hASCs)

The hASCs used in the experiment were obtained from Lonza (Cat. No. PT‐5006). These cells were cultured in ADSC basal medium (Lonza, Cat. No. PT‐3273), which was supplemented with the ADSC‐GM SinglequotsTM Supplement Kit (Lonza, Cat. No. PT‐4503). Cells were incubated at 37 °C at 5% CO2.

For dECM optimization, confluent cultures were treated once with 0.4% (w/v) OdECM and 0.3% CdECM, respectively, in culture media with media change every 3 days. To test for osteogenesis and chondrogenesis of GelO and GelC, 5 × 10^4^ were seeded in 24‐well plates at which the bottom of the wells was covered with a thin layer of GelO, GelC, or both.

For positive controls, Dulbecco′s Modified Eagle′s Medium (DMEM, Sigma, D5030) was used and supplemented with 10% FBS, 1% PS, 0.5 mM L‐ascorbic acid, and 10 mM BGF. To make an osteoinductive (OI) medium, 100 nM Dex was added and for a chondroinductive (CI) medium 100 nM Dex and 1% ITS were added to the media. All experimentations involving hASCs were carried out for 21 days.

### Scanning Electron Microscopy and Energy‐Dispersive X‐Ray Spectroscopy

The dECM and bioactive hydrogel samples underwent analysis using SEM/EDX following an established protocol,^[^ ^77^ ^]^ which combines scanning electron microscopy and energy‐dispersive X‐ray spectroscopy. A Hitachi SU3500 variable pressure SEM and an Oxford X‐Max50 SDD X‐ray detector were employed for this purpose. SEM generated surface topography images, while EDX, a semi‐quantitative technique, could detect elements ranging from carbon to uranium. Its minimum detection limit was ≈0.1 weight %, and it probed the sample to a depth of a few micrometres. The SEM/EDX analyses were conducted at an accelerating voltage of 20.0 kV. To minimize artifacts caused by sample charging, a thin layer of gold coating was applied to the samples.

### Cytocompatibility of the Hydrogels Using MTS Assay

The hASCs were seeded at a density of 15,000 cells per well in 96‐well plates and cultured overnight. In this study, two cytocompatibility tests were performed using cell proliferation MTS assay (Promega, Cat. No. G3580). This assay involved the conversion of MTS [3‐(4,5‐dimethyl‐2‐yl)−5‐(3‐carboxymethoxyphenyl)−2‐(4‐sulfophenyl)−2H‐tetrazolium] into formazan by living cells in a reducing environment. In the first experiment, the cells were exposed to different concentrations of CdECM and OdECM suspended in stem cell media (0.1, 0.3, 0.4, 0.5, and 0.6 (w/v)) for durations of 24 h. In the second experiment, thin layers of GelO and GelC were photo‐crosslinked with blue light (470 nm) on the bottom of 96‐well plates. They were washed with DI water and PBS. The same number of hASCs were seeded on coated wells and cultured overnight. After 24 h, 10 µL MTS reagent was added to each well of the 96‐well plate and incubated for 4 h in darkness. After incubation, the formazan supernatant was transferred to a fresh plate, and the absorption values were measured at a wavelength of 490 nm using a microplate reader.

### Quantitative Proteomics of OdECM and CdECM to Identify Their Protein Composition

A total phenotype of OdECM and CdECM was characterized by quantitative proteomic analysis using the Orbitrap Fusion Lumos mass spectrometer and interpreted using Spectronaut software. Functional enrichment analysis of identified proteins was conducted using the bioinformatics platform STRING database (http://string‐db.org) and enriched gene ontology terms under biological process, cellular components, and molecular function. The data were visualized using the bioinformatics platform SRplot with the help of R studio and the ggplot package.

### Preparation and Characterization of Hydrogels

GelMA was prepared using a previously established method.^[^ ^78^ ^]^ Initially, Gelatin from porcine skin (Type A) (Sigma, G2500) was dissolved in 10% PBS (w/v) at 60 °C under constant stirring for 1 h. After the complete dissolution of the gelatin, methacrylic anhydride (Sigma, 276685) (0.8 mL g^−1^ of gelatin) was slowly added dropwise to the gelatin solution while maintaining an alkaline pH. The reaction mixture was left to rotate for an additional 2 h at the same temperature. Subsequently, the GelMA solution was diluted with heated 100 mL PBS and subjected to dialysis for ≈1 week using a dialysis membrane with a molecular weight cut‐off of 12–14 kDa and distilled water maintained at 50 °C. The water used in the dialysis process was changed twice daily to remove any unreacted methacrylic anhydride, ensuring the elimination of potential toxicity. The resulting GelMA solution was then lyophilized (Labconco, USA) to obtain a powdered form. By varying the molar ratio of methacrylic anhydride to gelatin, the degree of methacrylation was adjusted to ≈73%.

Robust hydrogels containing 15% GelMA, 0.4% dECM, 1% Lithium phenyl‐2,4,6‐trimethylbenzoylphosphinate (LAP), and 0.1% tartrazine were fabricated on polydimethylsiloxane (PDMS) dishes using a digital light processing (DLP)‐based 3D printer following our previously designed fabrication strategy.^[^ ^79^ ^]^ Briefly, hydrogels were fabricated with a Lumen X DLP 3D printer (CELLINK, USA), which uses a light source at 405 nm and a power of 30–50 mW cm^−2^ for crosslinking. Printing parameters of 100 µm resolution, 80% power, 8 s of exposure time, and 4x exposure time for the first step were selected for printing our hydrogels. The computer‐aided design software (OnShape) was used to generate the digital models used in this study. After printing, the hydrogels were either immediately used or stored after lyophilization.

### Degradation of Hydrogels

To assess the impact of dECM particles on the degradation behavior, GelMA, GelO, and GelC hydrogels were prepared with a volume of 150 µL and lyophilized. The experiment involved collecting samples with 3 replicates on day 10. The hydrogels were placed in 24‐well plates and immersed in 2 mL of PBS containing 1% PS. The plates were placed in a shaker incubator with a temperature of 37.0 °C and a speed of 30 RPM. The hydrogels were freeze‐dried and weighed at the specified time point. The degradation rate (%) was determined using the following equation:

(1) $$DP=\frac{W_{0}-W}{W_{0}}\;*100\%$$

Equation 1: Degradation Percentage^[^ ^80^ ^]^

Here, W_0_ represents the initial weight of the dried hydrogel, W corresponds to the weight of the dried hydrogel at day 10, and DP is the degradation percentage.

### Swelling of Hydrogels

Swelling ratio of the hydrogels was determined following the established protocol.^[^ ^77^ ^]^ To evaluate the impact of dECM particles on the swelling characteristics of hydrogels, GelMA, GelO, and GelC hydrogels were prepared (n = 5) with a volume of 150 µL each. The dried weights of the hydrogels were recorded as W_0_. Then, dried hydrogels were placed into separate wells of a 24‐well plate containing 2 mL of PBS at a temperature of 37.0 °C. At intervals of 30 min for 7 h, the mass of each hydrogel was measured to calculate the swelling ratio using the following equation:

(2) $$S=\frac{W-W_{0}}{W_{0}}\;*100\%$$

Equation 2: Swelling Percentage^[^ ^80^ ^]^where S represents the swelling ratio expressed as a percentage, W represents the weight of the swollen gel, and W_0_ denotes the initial dry weight.

### Compressive Modulus of Hydrogels

To determine the compressive moduli of hydrogels, mechanical testing was conducted using INSTRON Force Transducer Model 2519‐101 under unconfined uniaxial conditions.^[^ ^81^ ^]^ Three groups of cylindrical hydrogels were 3D‐printed using DLP. The groups with 5 replicates were GelMA, GelO, and GelC with a diameter of 3 mm and height of 6 mm. To prevent slippage during the test, sandpaper was applied to the compression probes. The testing was performed on the swollen hydrogels at 0.3 mm min^−1^ compression rate, and the compressive modulus was determined by analyzing the linear portion of the stress–strain curve within the 1–15% strain range. The samples were compressed until fracture occurred. The compressive modulus profile was determined using *Equation* *3*, in which σ represents the applied compressive stress, and ε represents the strain.

(3) $$E_{comp}=\frac{\sigma_{comp}}{\varepsilon }\;$$

Equation 3: Compressive Modulus Formula

### Rheological Analysis of Hydrogels

The rheological studies were conducted using a HAAKE Modular Advanced Rheometer System (MARS) equipped with a P20/Ti titanium plate as the measuring geometry provided by Fischer Scientific, USA. The experiments were performed at 37 °C to simulate physiological conditions. Cylindrical GelMA, GelO, and GelC samples with dimensions of 6 mm × 6 mm were printed with three replicates. Before the rheological measurements, the hydrogels were hydrated in PBS at pH 7.4 for 5 min to prevent stickiness during testing. Any excess liquid on the gel surfaces was gently dabbed off to maintain uniformity. The rheological properties were evaluated through stress sweeps ranging from 0.1 to 104 Pa, applying different levels of stress to the hydrogel samples and measuring their corresponding strain responses.

### Carbodiimide‐Based Coupling Reaction for Adhering Gel‐O to Gel‐C

To chemically bond GelO and GelC hydrogels, an adhesive solution was synthesized using an established protocol.^[^ ^82^ ^]^ To prepare the adhesive solution, 2‐(N‐morpholino)ethanesulfonic acid (MES) sodium salt was dissolved in DI water at a concentration of 0.1 mol L^−1^ at room temperature, resulting in a 0.1 M MES buffer solution. The pH of the MES buffer solution was adjusted to 6 by gradually adding hydrochloric acid solution. Gelatin at a concentration of 20% (w/v) was dissolved in Eppendorf tubes using the MES buffer, and the tubes were then placed in a 60 °C environment for 10 min to aid in the dissolution process. N‐(3‐Dimethylaminopropyl)‐N’‐ethylcarbodimide Eppendorf tubes using the 0.1 M MES buffer at room temperature. Similarly, N‐Hydroxysuccinimide (NHS, Sigma, 130672–5G) at a concentration of 5% (w/v) was dissolved in Eppendorf tubes using the 0.1 M. The adhesive components were mixed right before applying between the two hydrogels and left to rest at room temperature for 1 h.

### Alizarin red S Staining (ARS) to Demonstrate Osteogenesis

The ARS method was employed to assess mineralization in cell culture samples and hydrogel constructs based on the manufacturer's protocol.^[^ ^83^ ^]^ Initially, confluent MC3T3‐E1 cells were cultured with growth media or ODM for 14 days. The monolayers were washed with PBS and fixed using a 4% paraformaldehyde solution to preserve the cellular and extracellular matrix components. Subsequently, the fixed samples underwent rinsing with deionized water to eliminate any residual fixative. To visualize the mineralized areas, the samples were stained with 40 mM Alizarin Red (ScienCell, Cat. No. 8678) at room temperature, which selectively binds to calcium deposits. After 30 min, the samples were thoroughly washed to remove excess dye and reduce background interference. The stained samples were then examined under a microscope to visualize the mineralized regions, indicated by the red staining (EDC, Sigma, E7750‐1G) at a concentration of 10% (w/v) was dissolved in. Quantification of mineralization was performed through colorimetric analysis by extracting the dye and measuring the absorbance at 405 nm wavelength. To ensure accurate quantification, calibration curves using known concentrations of calcium standards were utilized. Similarly, ARS was performed on MSCs that were grown on GelO for 21 days.

### Alcian Blue Staining to Demonstrate Chondrogenesis

Confluent ATDC5 cells were cultured with growth media or CDM for 14 days. The monolayers were washed with PBS and fixed using 95% methanol for 20 min. A solution of 1% Alcian Blue 8GX (Thermo Fisher, J60122) in 0.1 M HCl was prepared. After washing off the methanol, the monolayers were exposed to the dye solution overnight. Following this, the samples were thoroughly washed with PBS and then deionized water. Images of the stained cells were captured. Similarly, ABS was performed on MSCs that were grown on GelC for 21 days.

### Sulfated‐Glycosaminoglycans (S‐GAG) Quantification to Prove chondrogenesis

To evaluate chondrogenesis by GelC, hASCs cultured on a thin layer of hydrogel for 21 days were subjected to Dimethylmethylene Blue Assay (DMMB, ABCAM, ab289846) based on the manufacturer protocol. Briefly, 100 mg of Cell/hydrogel samples were transferred to Eppendorf tubes, followed by the addition of ice‐cold homogenization buffer. The resulting mixture was centrifuged at 12 000 x g and 4 °C for 20 min. The supernatant, obtained after centrifugation, was collected for subsequent analysis. In a 96‐well plate, 50 µL of the supernatant was added to a well labelled as “Sample,” while another well labelled as “Sample Control” received 50 µL of homogenization buffer. Both wells were adjusted to 100 µL using S‐GAG Assay Buffer. To create the standard curve, a diluted s‐GAG Standard solution was prepared by combining s‐GAG stock Standard with s‐GAG Assay Buffer. Subsequently, 200 µL of s‐GAG Dye was added to all wells. After a 2‐min incubation at room temperature, the absorbance of all wells was measured at 525 nm.

### DAPI/Phalloidin Staining to Determine Cell Morphology

DAPI/phalloidin staining technique was employed to visualize nuclear DNA and filamentous actin. Samples were washed with PBS followed by the addition of 4% paraformaldehyde for a 5‐min incubation at room temperature. Cells were then treated with 0.1% Triton X and incubated at room temperature for 10 min. After three washes with PBS, a 1:40 dilution of phalloidin was added to the samples and incubated at room temperature for 30 min. Following another round of three washes with PBS, a 1:1000 dilution of DAPI (Thermo Fisher, D1306) was added and incubated at room temperature for 3 min. Subsequently, the samples were washed with PBS and mounted. The mounted samples were subjected to fluorescence microscopy to examine the morphology of actin filaments, which were pivotal in regulating cell shape, polarity, and crucially, cell motility. To assess the successful decellularization of differentiated ECM, DAPI staining was performed on CdECM and OdECM after 14 days.

### Reverse Transcription‐Quantitative Polymerase Chain Reaction (RT‐qPCR) Analysis to Quantify Gene‐Markers Associated with Osteogenesis and Chondrogenesis

RT‐qPCR analysis was carried out for hASCs grown on 24‐well plates and treated with 0.3% CdECM, 0.4% OdECM, GelO, GelC, or GelO‐GelC for 21 days. RNA isolation was performed using the RNeasy mini kit (Qiagen, Cat. No. 74104) according to the manufacturer's protocol. Briefly, cells were harvested from the monolayer and lysed using 350 µL of lysis buffer, ensuring the release of RNA while preserving its integrity. The lysate was then mixed with 70% ethanol to facilitate RNA binding to the silica membrane of the RNeasy spin column. The column was subsequently washed to remove impurities, and the bound RNA was eluted in a separate collection tube. The extracted RNA was quantified and assessed for quality using NanoDrop (Thermo Scientific, Canada). Five hundred nanograms of total RNA were used to synthesize cDNA using a High‐Capacity cDNA Reverse Transcription Kit (Thermo Fisher, Cat. No. 4368814). Reverse transcription was performed in a Chromo4TM Real‐Time PCR Thermocycler (BioRad, Canada).

TB Green Advantage qPCR premix (Takara Bio, Cat. No. 639676) was used for the amplification of osteogenic and chondrogenic markers. The PCR reaction mixture was prepared by combining the TB Green Advantage qPCR Premix, PCR forward and reverse primers (See **Table** **1** for primer sequences), template cDNA, and nuclease‐free water according to the recommended volumes and concentrations provided. The target osteogenic markers were Runt‐Related Transcription Factor 2 (*RUNX2)*, osteocalcin (*OCN*), and osteopontin (*OPN*); the chondrogenic markers were aggrecan (*ACAN*), SRY‐Box Transcription Factor 9 (*SOX9*), and Matrix metalloproteinase 13 (*MMP13*); and the reference gene was *GAPDH* (housekeeping gene). After gentle centrifugation, the samples were placed in the LightCycler 96 System (Roche, Canada). The reaction was initiated with an initial denaturation step at 95 °C for 10–30 s with detection turned off. Subsequently, combined annealing and extension were carried out at a temperature range of 55 to 72 °C for 15 s with detection turned on. The cycle number was set to 45 cycles. Following the completion of the reaction, the amplification curve and melting curve were analyzed for further assessment. After establishing a standard curve for each primer set, quantification of gene expression was performed using the Pfaffl Method^[^ ^84^ ^]^ with *GAPDH* and control groups being the reference gene and the calibrator, respectively.

**Table 1:** RT‐qPCR forward and reverse primer sequences.

Genes	Forward Primer (5′>3′)	Reverse Primer (3′>5′)	T_m_ (°C)
*GAPDH*	AACAGCGACACCCACTCCTC	CATACCAGGAAATGAGCTTGACAA	63
*SOX9*	GGCAAGCTCTGGAGACTTCTG	CCCGTTCTTCACCGACTTCC	62
*ACAN*	AGTGCACAGAGGGGTTTGTC	CGTTTGTAGGTGGTGGGGTC	60
*MMP13*	TCCAGTCTCTCTATGGTCCAGG	CCTCGGAGACTGGTAATGGC	61
*OCN*	CACCGAGACACCATGAGAGC	CTGCTTGGACACAAAGGCTGC	65
*OPN*	GTGCAGAGGAAACCGAAGAG	TGTTTGCAGTGGTGGTTCTG	61
*RUNX2*	TCTCCAGGAGGACAGCAAGA	CTGCTTGCAGCCTTAAATGACT	61

### Statistical Analysis

The experimental data were expressed as the mean ± standard deviation. Differences between the experimental groups were assessed using statistical tests appropriate for the group size: the Student's *t*‐test for two groups, one‐way ANOVA for three or more groups, followed by Tukey post hoc comparisons. Statistical significance was defined as a p‐value less than 0.05. ^(*^ = *p*<0.05, ^**^ = *p*<0.01, ^***^ = *p*<0.001, ^****^ = *p*<0.0001).

## Conflict of Interest

The authors declare no conflict of interest.

## Supporting information



Supporting Information

## Data Availability

The data that support the findings of this study are openly available in bioRXiV at https://doi.org/10.1101/2024.03.04.583366, reference number 583366.
