# Supplementary material for: In Vitro Engineered ECM‐incorporated Hydrogels for Osteochondral Tissue Repair: A Cell‐Free Approach
Source: Adv Healthc Mater. 2025 Jan 5;14(4):2402701. doi: 10.1002/adhm.202402701 (PMC11804842; doi:10.1002/adhm.202402701)
Supplement: Supplementary file 1 — Supporting Information [file ADHM-14-0-s001.pdf]

ADVANCED HEALTHCARE MATERIALS

Supporting Information

for *Adv. Healthcare Mater.*, DOI 10.1002/adhm.202402701

In Vitro Engineered ECM-incorporated Hydrogels for Osteochondral Tissue Repair: A Cell-Free Approach

*Ali Coyle, Aishik Chakraborty, Jiaqi Huang, Yasmeen Shamiya, Wei Luo and Arghya Paul**

Supporting Information

In vitro Engineered ECM-incorporated Hydrogels for Osteochondral Tissue Repair: A Cell-free Approach

Ali Coyle[1,†], Aishik Chakraborty[2,3,†], Jiaqi Huang[2], Yasmeen Shamiya[4], Wei Luo[1], Arghya Paul[1,2,3,4,*]

1. School of Biomedical Engineering, The University of Western Ontario, London, ON N6A 5B9, Canada

2. Department of Chemical and Biochemical Engineering, The University of Western Ontario, London, ON N6A 5B9, Canada

3. Collaborative Specialization in Musculoskeletal Health Research and Bone and Joint Institute, The University of Western Ontario, London, ON N6A 5B9, Canada

4. Department of Chemistry, The University of Western Ontario, London, ON N6A 5B9, Canada

* Corresponding author

† equal contribution

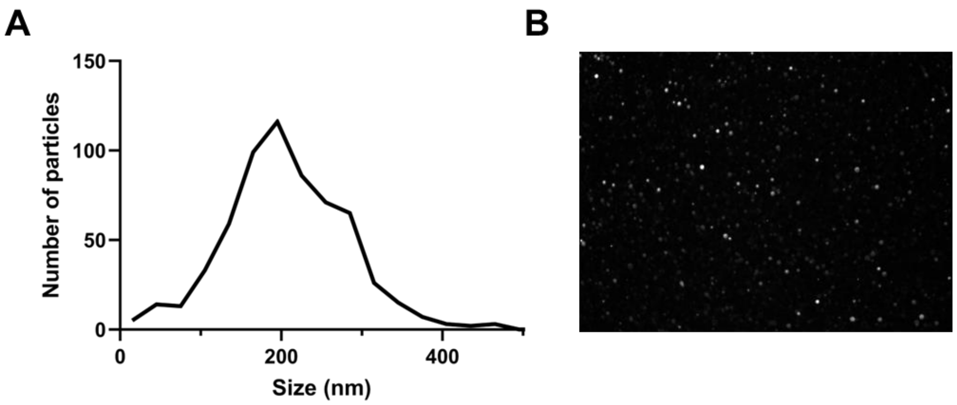

Figure S1: Size distribution of dECM. A) The graph displays the size distribution of our dECM particles measured using Nanoparticle Tracking Analysis, Zetaview PMX-130 (Particle Metrix, Germany). **B)** Representative light scatter image demonstrates particle suspension. This image was captured using a 10x objective microscope coupled with a video camera.

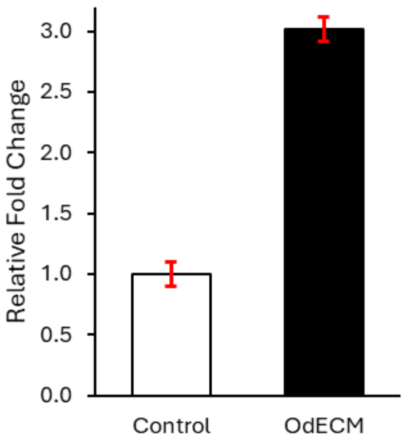

Figure S2: RT-qPCR demonstrating the upregulation of Runx2. The figure displays a 3-fold upregulation of Runx2 when hASCs were differentiated with OdECM.

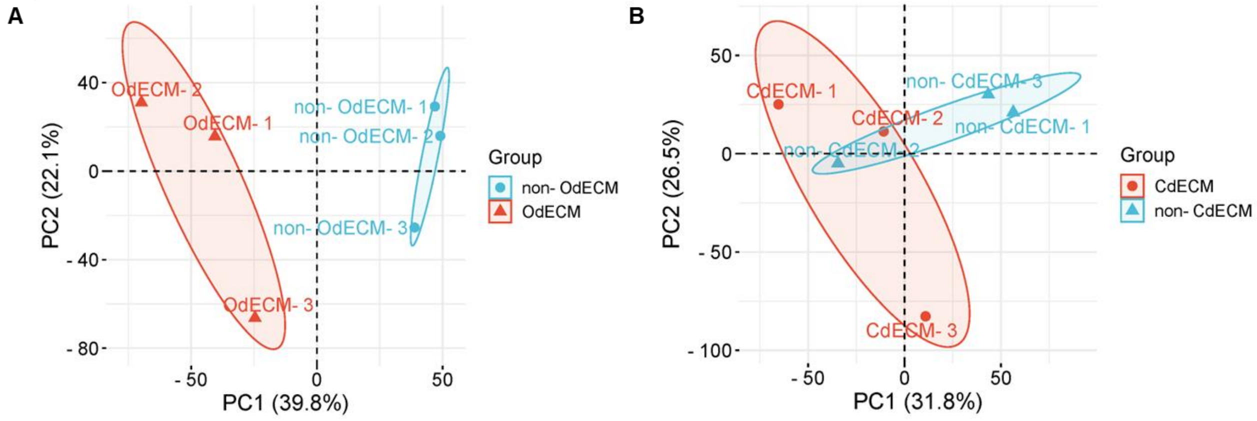

Figure S3. Principal component analysis (PCA) of the samples based on proteomics data displaying variance within the dataset. A) PCA profile of OdECM/ non-OdECM samples. **B)** PCA profile of CdECM/non-dECM samples.

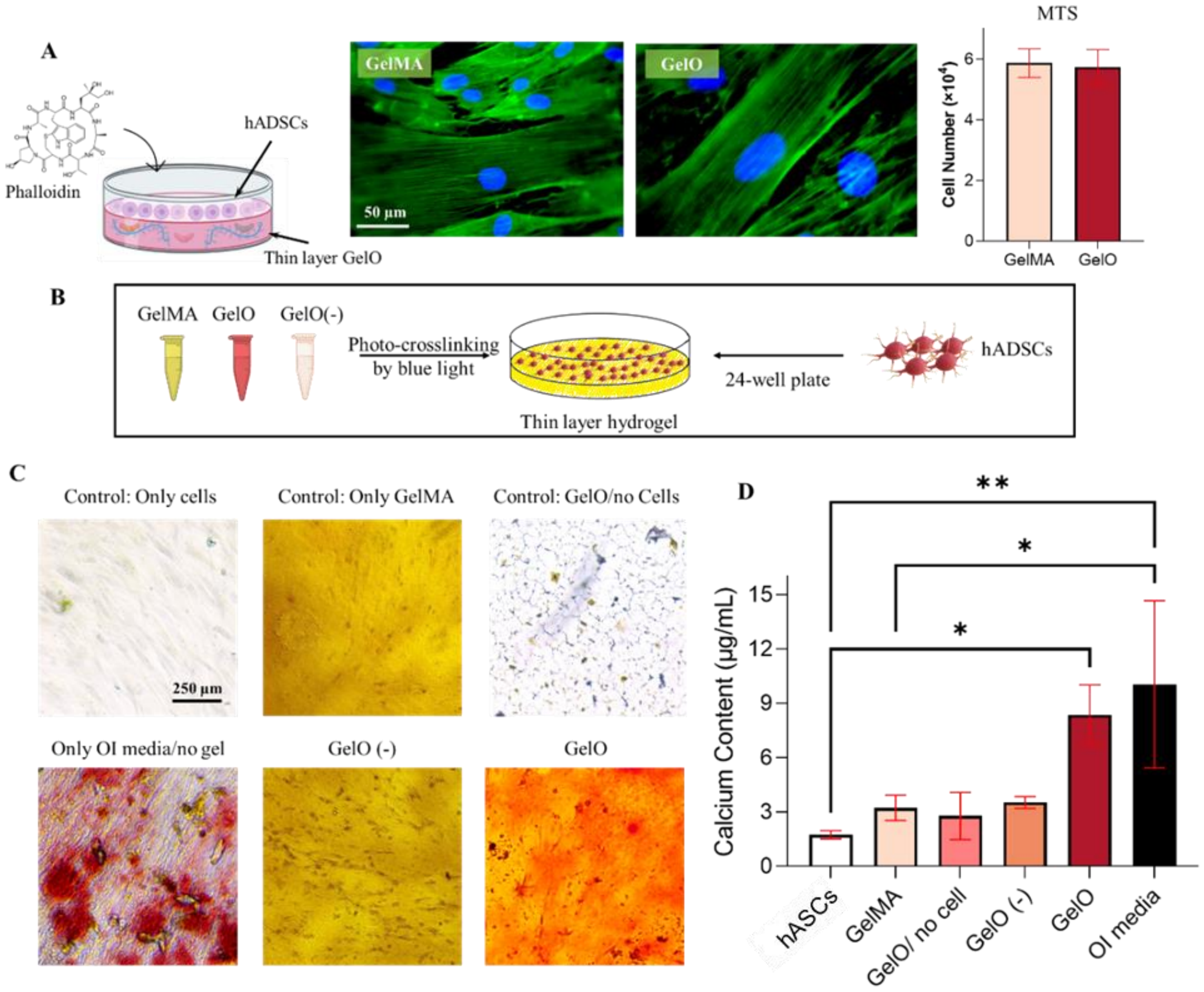

Figure S4. GelMA containing bioactive OdECM (GelO) can induce drug and cell-free osteogenic differentiation in hASCs. A) Phalloidin/DAPI Staining and MTS Assay Results: Fluorescent staining using Phalloidin (green) and DAPI (blue) was performed on hASCs cultured on thin layer GelMA and GelO for 24 hours. The staining revealed that the organization and morphology of the cytoskeleton and actin filament (green) and cell nuclei (blue) stayed intact. The corresponding MTS assay was conducted to assess the metabolic activity and cell viability of the stem cells. The bar graph represents the cell number, demonstrating the relative cell viability between the two groups. **B)** Alizarin Red Staining Experiment on hASCs: An illustration depicts the experimental setup for the Alizarin Red staining assay performed on hASCs. The cells were cultured on a thin layer of hydrogels for 21 days to induce osteogenic differentiation. **C)** Microscopy Images of Alizarin Red Stained hASCs: Microscopy images exhibit the results of Alizarin Red staining on hASCs. The sample treated with osteoinductive media (OI), containing dexamethasone in DMEM, acted as positive group while all the other groups were cultured in ADSC-BM media. GelO samples showed visible mineralization that indicates successful osteogenic differentiation. The GelO (-) group refers to the hydrogel that contains 0.4% (w/v) dECM derived from non-differentiated MC3T3-E1-E1 cells. **D)** Quantification of Alizarin Red Staining: Quantitative analysis was performed to measure the dye extraction from the Alizarin Red staining assay. The results demonstrated that hASCs treated with GelO exhibited significantly higher levels of staining compared to controls, suggesting enhanced osteogenic differentiation.

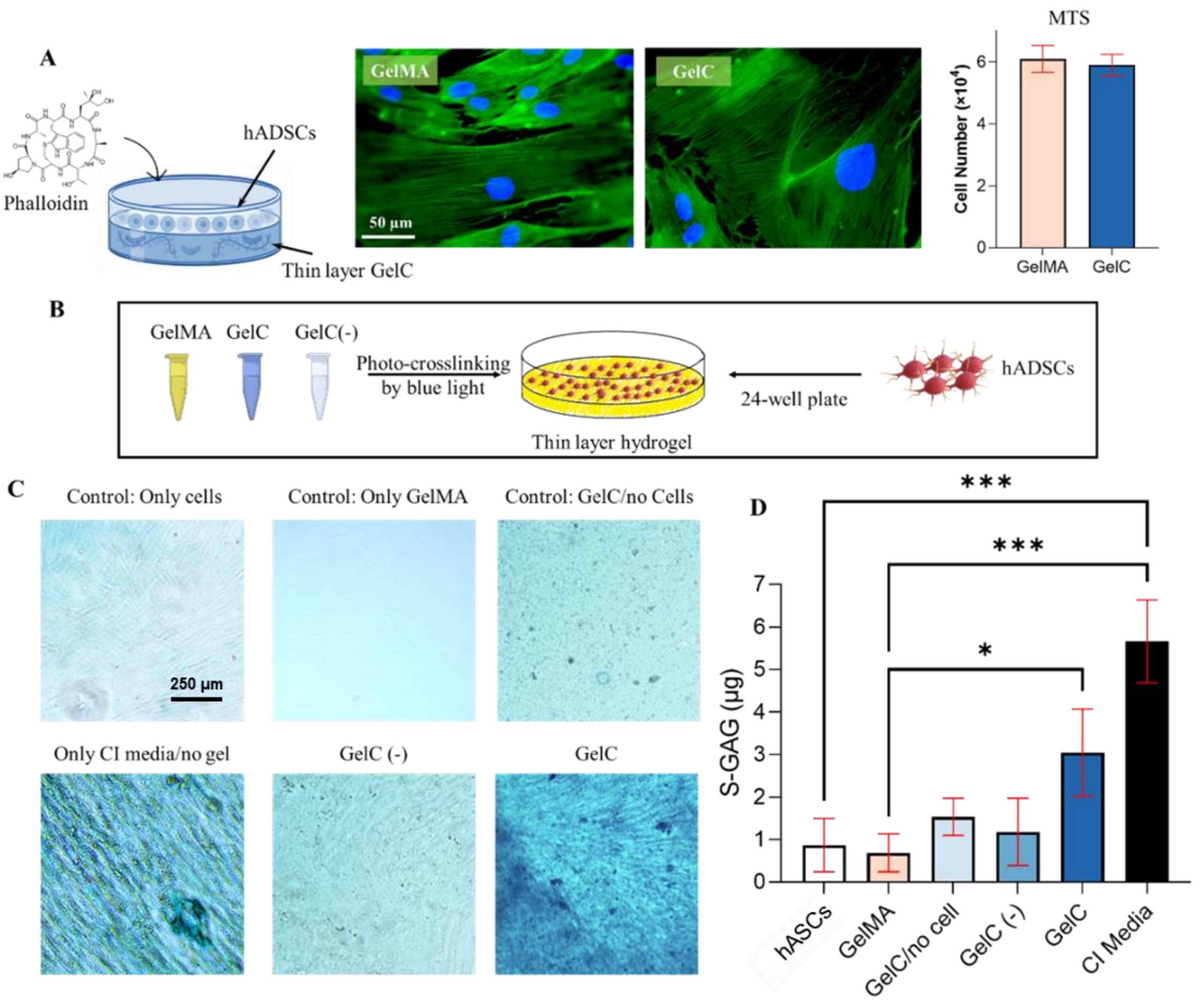

Figure S5. GelMA containing CdECM (GelC) can induce drug and cell-free chondrogenic differentiation in hASCs. A) Phalloidin/DAPI Staining and MTS Assay Results: Phalloidin/DAPI staining was performed on stem cells cultured on GelMA and GelC after 24 hours to visualize the preservation of cytoskeleton (green) and cell nuclei (blue). The corresponding MTS assay was conducted to assess the metabolic activity and cell viability of the stem cells. The bar graph presents the cell number values obtained, indicating similar cell viability between the two groups. *Note: The fluorescence image for GelMA and the corresponding MTS data is the same as in* ***Figure 4A****. This is because GelMA serves as the control for both GelO and GelC.* **B) Illustration of Alcian Blue Staining Experiment on hASCs**: An illustration depicts the experimental setup for the Alcian Blue staining experiment conducted on hASCs grown on a thin layer of GelC for 21 days to induce chondrogenic differentiation. **C) Microscopy Images of Alcian Blue Stained hASCs**: Microscopy images display the results of Alcian Blue staining on hASCs. The samples treated with chondroinductive media (CI), containing dexamethasone and ITS serum in DMEM/F12, acted as positive group while all the other groups were cultured in *h*ASC-BM media. GelC samples exhibited positive staining for sulfated glycosaminoglycans (s-GAG), indicating successful chondrogenic differentiation. GelC (-) represents the that contains 0.3% (w/v) dECM derived from non-differentiated ATDC5 cells. **D) Quantification of s-GAG**: Quantitative analysis was conducted to measure the sulfated glycosaminoglycan (s-GAG) content with the use of Sulfated-Glycosaminoglycans Assay Kit. hASCs treated with GelC hydrogel demonstrated significantly higher levels of s-GAG compared to the control groups, suggesting enhanced chondrogenic differentiation.
